# Depletion of p75NTR in Schwann Cells Driven by Inflammation Mediates Cutaneous Pain in Psoriasis

**DOI:** 10.1002/advs.202523189

**Published:** 2026-04-03

**Authors:** Yibo Wang, Linlin Xu, Chenglong Pan, Ruonan Cao, Piao Zeng, Xinxing Lyu, Qingxia Hu, Zhenzhen Yan, Shuhong Huang, Ningning Dang

**Affiliations:** ^1^ Department of Dermatology Shandong Provincial Hospital Affiliated to Shandong First Medical University Jinan Shandong China; ^2^ School of Clinical and Basic Medicine Shandong First Medical University & Shandong Academy of Medical Sciences Jinan Shandong China; ^3^ Hospital For Skin Diseases Shandong First Medical University Jinan Shandong China

**Keywords:** cutaneous pain, NGF, p75NTR, psoriasis, schwann cell

## Abstract

Skin pain is a common but poorly understood symptom of psoriasis, affecting only a subset of patients. Using imiquimod and interleukin‐17A‐induced psoriasiform mouse models that exhibited pain‐like behaviors, we found that nerve growth factor (NGF) levels were elevated in lesional skin, activating TrkA signaling in dorsal root ganglion neurons and promoting Schwann‐cell hypertrophy. Normally, Schwann cells (SCs) limit NGF signaling in cutaneous peripheral nerves through the p75NTR receptor. However, inflammation driven by interleukin‐17A increased non‐muscle myosin II activity and elevated NGF levels, leading to the internalization and degradation of p75NTR. The resulting depletion of p75NTR caused local NGF accumulation, excessive TrkA activation, and heightened pain sensitivity. These findings reveal that psoriatic inflammation converts SCs from protective buffers into drivers of pain, offering a mechanistic explanation for why only some patients experience cutaneous pain in psoriasis.

## Graphical abstract

Psoriasis‐like inflammation induces proliferation and molecular remodeling of cutaneous Schwann cells, marked by reduced p75NTR and increased NGF expression. IL‐17A promotes this process, whereas Schwann cell‐specific p75NTR overexpression alleviates cutaneous pain in vivo. These findings identify a Schwann cell‐dependent mechanism of pain sensitization and suggest p75NTR as a potential therapeutic target for psoriatic pain.

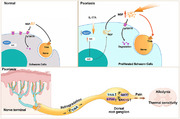

## 1 Introduction

Psoriasis is a chronic, relapsing systemic inflammatory skin disease that affects ∼ 2%–3% of the global population [1, 2]. Although pruritus is the dominant symptom—reported by > 80% of patients—roughly one‐third also experience clinically meaningful skin pain (mean intensity ≈ 6/10), which markedly reduces quality of life and correlates with higher Dermatology Life Quality Index (DLQI) scores [3, 4, 5]. Despite its clinical importance, the cellular and molecular mechanisms that produce pain in only a subset of patients remain poorly defined.

Psoriatic pain is best classified as nociceptive, with intertwined immune and neural drivers [6]. Studies have documented increased substance P in epidermal nerve fibers of psoriatic lesions [7]. The IL‐23/IL‐17 immune axis underpins psoriasis pathogenesis, and in the imiquimod (IMQ) model dermal dendritic cells that secrete IL‐23 localize near nociceptive neurons, linking IL‐23–driven inflammation to sensory changes [8, 9]. Histologically, lesional hyperinnervation commonly accompanies these immune alterations.

Work on keratinocytes and fibroblasts has clarified how cutaneous non‐neuronal cells reshape sensory innervation in psoriasis [10]. Analogous to central nervous system (CNS) astrocytes, peripheral glia—including satellite glia and repair Schwann cells (SCs)—react to injury or inflammation and modulate neuronal excitability, mechanisms implicated in chronic pain [11]. Recently, a distinct population of nociceptive Schwann cells was described at the dermal–epidermal junction; these cells form a reticular glial network with radial projections that interact with unmyelinated fibers to create glial–neurite organoids [12]. Although these data implicate Schwann‐cell subtypes in cutaneous nociception, the roles of Schwann cells specifically within psoriatic lesions remain largely unexplored.

Nerve growth factor (NGF) is a central modulator of peripheral nociception: clinical and experimental studies link elevated NGF to inflammatory pain states, including osteoarthritis [13]. Peripheral nerve terminals transduce stimuli and relay excitatory signals to dorsal root ganglion (DRG) neurons, whose somata integrate somatosensory input and transmit afferent information to the spinal cord and higher centers [14]. Multiple reports have documented increased NGF expression in psoriatic tissue [15].

NGF signals primarily via two receptors, the high‐affinity receptor TrkA and the low‐affinity pan‐neurotrophin receptor p75NTR [16]. TrkA is highly expressed on cutaneous nociceptor terminals and DRG neurons, whereas p75NTR serves as a canonical marker of peripheral glia (Schwann cells) and contributes to axon guidance, myelin remodeling, and neural homeostasis [17]. Although the NGF–TrkA axis has established links to pain, investigators have not systematically defined how Schwann‐cell p75NTR governs cutaneous NGF signaling. When co‐expressed with TrkA, p75NTR can enhance NGF affinity for TrkA and thereby modulate downstream signaling; recent work also implicates p75NTR in proBDNF‐driven pain [18], but those studies focused on neuronal p75NTR rather than glial expression.

Here, we ask whether Schwann cells regulate NGF‐evoked nociceptive signaling in psoriatic skin and whether inflammatory mediators convert Schwann cells from protective buffers into drivers of pain. We show that IL‐17A and NGF jointly induce Schwann‐cell proliferation and morphological remodeling that initially restrain NGF signaling via p75NTR, but that sustained inflammatory stress promotes p75NTR internalization and degradation. This p75NTR depletion increases local NGF availability, enhances DRG TrkA activation, and drives cutaneous nociception. These findings establish Schwann cells as active modulators of pain in psoriasis and provide a mechanistic link between local inflammation and interindividual variability in psoriatic pain.

## 2 Results

### 2.1 Psoriasis Evokes Coping Behavior Associated With Pain

**FIGURE 1:**
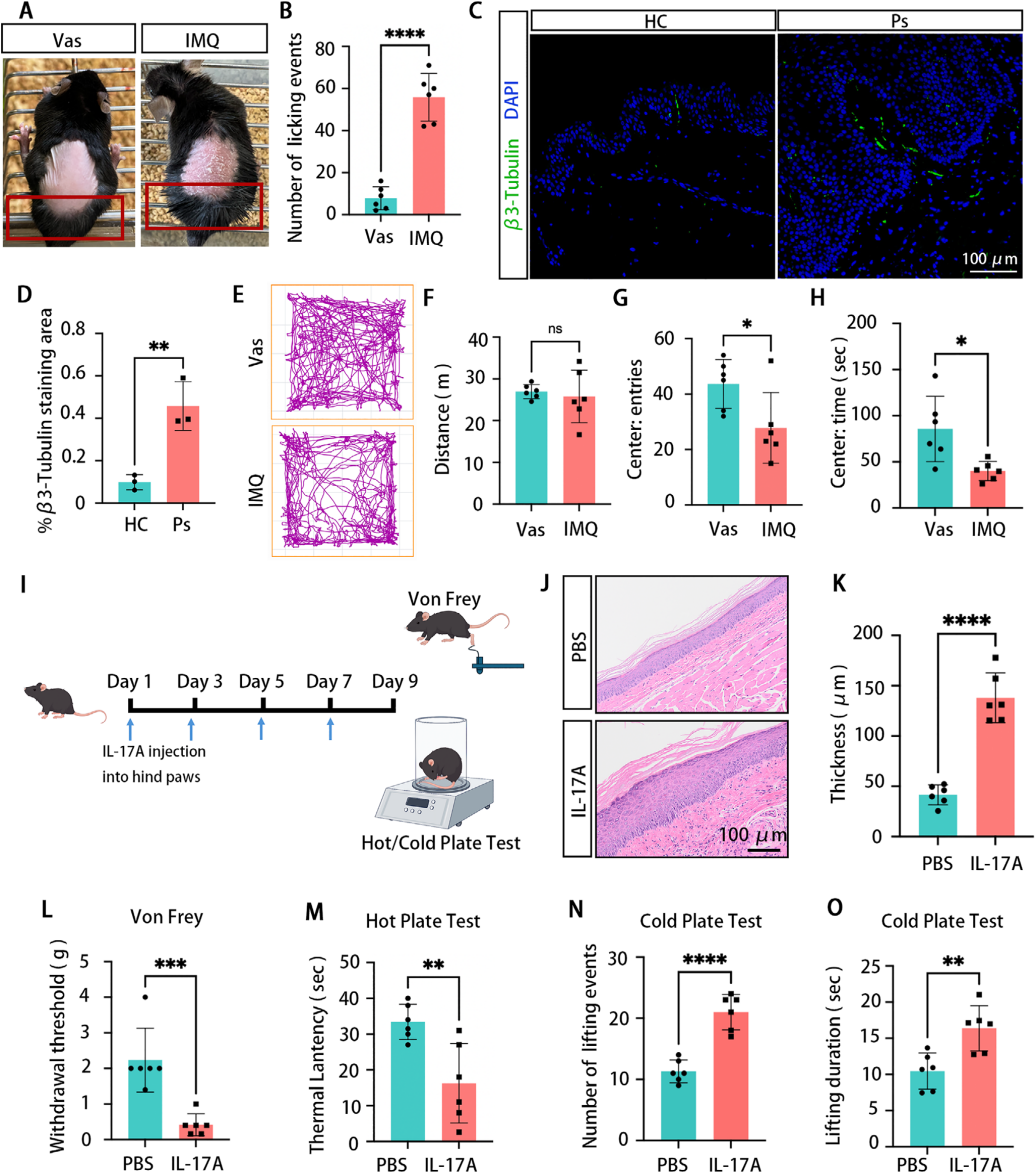
Psoriasis evokes pain‐associated coping behaviors. (A) Representative images of dorsal skin and surrounding hair in Vas‐ and IMQ‐treated mice. Red‐boxed areas are magnified to highlight changes in fur around lesions. (B) Quantification of licking events over 1 h. (C) Representative immunofluorescence images of β3‐tubulin in healthy controls (HC) and psoriasis patients (Ps). (D) The %β3‐tubulin‐positive staining area in C. (E) Representative movement tracks of mice in the open field arena during a 10‐min session. (F) Total distance traveled (m) in the open field. (G) Number of entries into the central zone. (H) Duration spent in the central zone (s). (I) Diagram of the IL‐17A‐induced psoriasis model in C57BL/6 mice injected in hind paws with PBS or IL‐17A (created using Figdraw). (J) H&E staining of PBS‐ and IL‐17A‐injected hind paw skin. (K) Quantification of epidermal thickness in PBS‐ versus IL‐17A‐injected skin. (L) Paw withdrawal threshold (g) measured using Von Frey filaments. (M) Latency to jumping (s) on a hot plate (55°C). (N) Number of hind paw lifts during 5‐min cold plate exposure (4°C). (O) Total duration of hind paw lifting (s) during cold plate exposure. Scale bars as indicated. ns, not significant; *p < 0.05, **p < 0.01, ***p < 0.001, and ****p < 0.0001; according to unpaired Student's *t*‐test (B, D, F, G, H, K, L, M, N, O). *n* = 3 (D), *n* = 6 (B, F, G, H, K, L, M, N, O).

Skin pain is a common but underappreciated symptom of psoriasis [4]. To model this, we used the widely adopted IMQ‐induced psoriasiform mouse model [19]. IMQ‐treated mice frequently licked their dorsal lesions, producing wet, matted fur around affected sites (Figure 1A); this licking—distinct from the biting typical of pruritus—reflects a nocifensive response to pain [20]. Quantification confirmed a significant increase in dorsal‐licking events in IMQ‐treated animals versus Vas‐treated controls during a 1‐h observation (Figure 1B), indicating nociceptive behavior.

Because increased cutaneous innervation often accompanies pain [21], we examined nerve density by immunofluorescence and observed a marked increase in intra‐lesional nerve fibers in IMQ‐treated mice (Figure S1A,B). Consistently, the nerve area was also increased in the skin of patients with psoriasis (Figures 1C,D). Systemic sequelae of pain, such as weight loss and anxiety‐like behavior [22, 23], also emerged in IMQ‐treated mice: body weight decreased significantly relative to controls (Figure S1C), and open‐field testing, which is an ethological test for measuring anxiety‐like behavior in mice [24] revealed no locomotor deficit (Figure 1E,F) but showed fewer center entries and reduced time in the center zone (Figure 1G,H), consistent with anxiety. Together, these behavioral and histological data show that IMQ‐treated mice recapitulate pain‐associated phenotypes.

IL‐17A drives psoriasis‐like inflammation and cutaneous hypersensitivity. IL‐17A is a central mediator of psoriasis pathogenesis and potently stimulates keratinocyte proliferation [25]. Because intradermal IL‐17A can induce psoriasis‐like lesions [26], we tested whether IL‐17A alone produces pain sensitization using a peripheral inflammation protocol (Figure 1I). We injected IL‐17A (1 µg, 20 µL) into both hind paws every other day, with PBS as a control. H&E staining confirmed model induction: epidermal thickness increased nearly threefold in IL‐17A–injected skin versus PBS (Figures 1J,K). Behavioral assays revealed robust sensory sensitization: IL‐17A–treated mice showed reduced mechanical withdrawal thresholds in the von Frey test (Figure 1L), shorter latencies on the hot plate (Figure 1M), and, after a 5‐min habituation on a 4°C cold plate, increased paw‐lift frequency and longer cumulative paw‐lift duration during the subsequent 5‐min observation (Figures 1N,O). Together, these data indicate that IL‐17A–driven cutaneous inflammation produces mechanical and thermal hypersensitivity characteristic of psoriatic pain.

### 2.2 Psoriatic Lesions Exhibit Elevated NGF With Cutaneous TrkA Loss and DRG Accumulation

**FIGURE 2:**
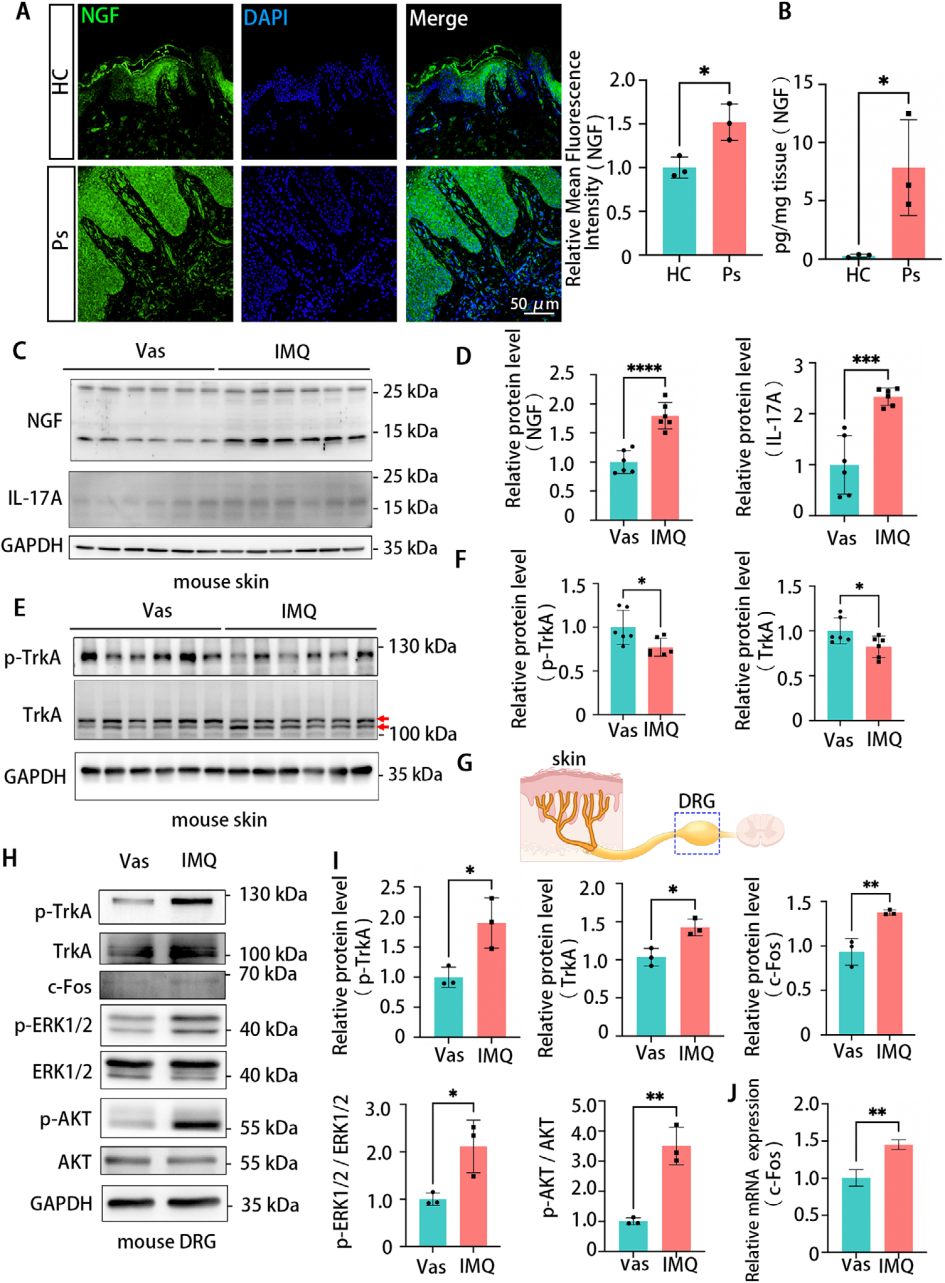
Psoriatic lesions exhibit elevated NGF with cutaneous TrkA loss and DRG accumulation. (A) Representative fluorescent immunohistochemistry images and quantification of NGF mean fluorescence intensity of NGF in healthy controls (HC) and psoriasis patients (Ps). (B) Quantification of NGF levels in skin tissues from healthy controls (HC) and psoriasis patients. (C) Western blot analysis of NGF and IL‐17A expression in dorsal skin of Vas‐ and IMQ‐treated mice. (D) Statistical analysis of NGF/IL‐17A expression from C. (E) Western blot analysis of p‐TrkA and TrkA protein expression in dorsal skin of Vas‐ and IMQ‐treated mice. (F) Quantification of p‐TrkA and TrkA expression from E. (G) Schematic of neurons with cell bodies in the dorsal root ganglia (DRG) projecting axons to the skin. (H) Western blot analysis of protein expression in DRG of Vas‐ and IMQ‐treated mice. (I) Quantification of protein expression from H. (J) Relative c‐Fos mRNA expression in DRG from Vas‐ and IMQ‐treated mice. In (E), the two arrow‐indicated bands together represent the total TrkA signal in mouse skin and were quantified in (F). Scale bar as indicated. *p < 0.05, **p < 0.01, ***p < 0.001, and ****p < 0.0001; unpaired Student's *t*‐test (A, B, D, F, I, J); *n* = 3 (A, B, I, J), *n* = 6 (D, F).

NGF critically initiates and sustains nociceptive signaling [27]. We observed elevated NGF levels in skin from patients with psoriasis, as demonstrated by both immunofluorescence staining and ELISA (Figure 2A,B). Western blotting revealed substantially elevated NGF in lesional skin of IMQ‐treated mice, with concordant increases in IL‐17A protein (Figure 2C,D). Paradoxically, total TrkA and phosphorylated TrkA protein levels in lesional skin were reduced by Western blot (Figure 2E,F), despite the known enrichment of TrkA on cutaneous nociceptor terminals and DRG neurons (Figure 2G). Given the concurrent rise in NGF and nerve density, we hypothesized that ligand‐driven TrkA internalization at peripheral terminals, followed by retrograde trafficking to the DRG, amplifies somatic signaling. To this end, we assessed protein expression in mouse DRG. DRG from IMQ‐treated mice contained higher TrkA and phosphorylated TrkA levels and showed increased activation of MAPK/ERK1–2 and PI3K/AKT pathways together with elevated c‐Fos (Figure 2H,I,J). Collectively, these observations are consistent with enhanced NGF–TrkA retrograde signaling and increased pain‐related signaling in DRG neurons, in line with previous findings in the rat sciatic nerve showing that NGF promotes retrograde TrkA transport [28].

### 2.3 IL‐17A and NGF Associate With MLC2‐Linked Cytoskeletal Remodeling in Schwann Cells

**FIGURE 3:**
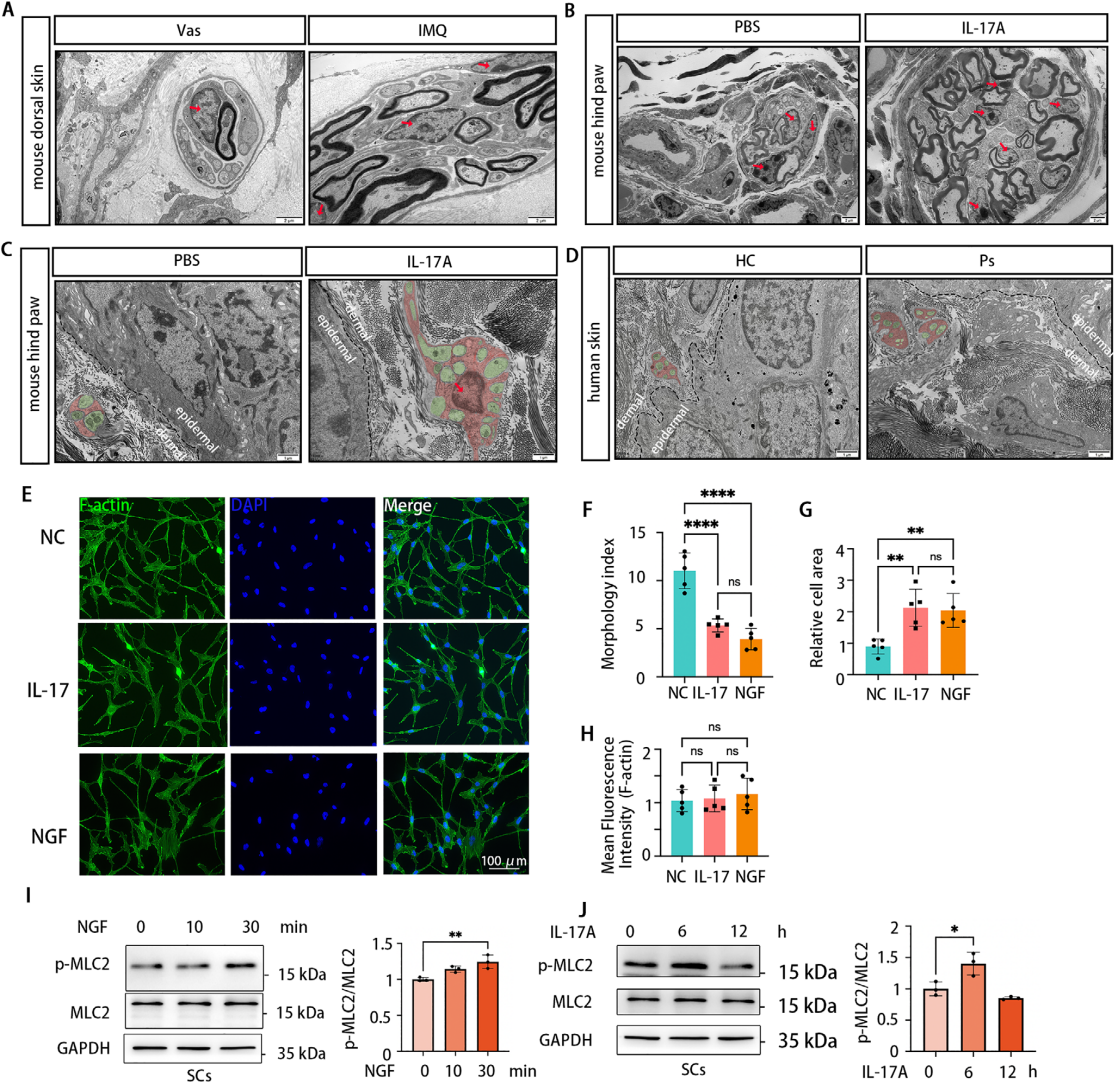
IL‐17A and NGF are associated with MLC2‐Linked cytoskeletal remodeling in Schwann cells. (A) Representative TEM images of myelinated bundles in the dorsal skin of Vas‐ and IMQ‐treated mice. (B) TEM images of myelinated bundles in PBS‐ or IL‐17A‐injected hind paws. (C) TEM images of glio‐neural complexes in PBS‐ or IL‐17A‐injected hind paws. (D) TEM images of glio‐neural complexes in healthy controls (HC) and psoriasis patients (Ps). Red arrows indicate Schwann cell nuclei in (A, B). Glio‐neural complexes at the dermal–epidermal junction were pseudo‐colored: axons (green), Schwann cells and processes (red) in (C, D). (E) Representative fluorescence images of Schwann cells under PBS (control) or IL‐17A/NGF treatment, stained with phalloidin. (F–H) Quantification of Schwann cell morphology index (F), relative cell area (G), and F‐actin mean intensity (H) under control conditions. (I, J) Schwann cells were stimulated with NGF (100 ng/mL, I) or IL‐17A (20 ng/mL, J) at the indicated time points, and total MLC2 and p‐MLC2 levels were analyzed by Western blot. Scale bars as indicated. ns, not significant; *p < 0.05, **p < 0.01, ***p < 0.001, ****p < 0.0001; one‐way ANOVA with post hoc Tukey (F–H), n = 5 (F–H), *n* = 3 (I, J).

Pain perception depends on precise neuron–glia interactions, and Schwann cells, peripheral glia that support axons, critically maintain nerve‐fiber function [29, 30]. To characterize structural alterations in lesional nociceptors, we used transmission electron microscopy. IMQ‐treated skin displayed an increased number of Schwann‐cell nuclei (Figure 3A), and IL‐17A–injected hind paw skin showed a similar Schwann‐cell expansion (Figure 3B). We observed Schwann cell–neuron complexes at the dermoepidermal junction (Figure 3C) and noted tighter Schwann‐cell enwrapping of axons in psoriatic lesions; comparable Schwann cell–axon rearrangements were present in human psoriatic samples (Figure 3D).

Because NGF and IL‐17A are elevated in lesions, we examined Schwann cell morphology using immunofluorescence. Schwann cells (identified by the Schwann cell‐specific marker SOX10 and S100β) (Figure S2A), treated with IL‐17A or NGF underwent pronounced morphological remodeling (Figure 3E), manifested as a lower morphology index (Figure 3F) and larger cell area (Figure 3G). Morphological remodeling was quantified using a morphology index, defined as the ratio of the maximum to minimum cell diameter, with higher values indicating a more elongated cell shape [31]. Phalloidin staining showed no change in total F‐actin fluorescence (Figure 3H), indicating that these shape changes reflect spatial reorganization of the actin cytoskeleton rather than net actin synthesis or degradation.

Myosin light chain 2 (MLC2), the regulatory light chain of non‐muscle myosin II (NMII), increases NMII ATPase activity when phosphorylated, promoting cross‐linking and sliding of F‐actin bundles and thereby altering cell mechanics [32]. We therefore tested whether IL‐17A/NGF–induced spreading correlates with MLC2 phosphorylation. Western blotting demonstrated increased p‐MLC2 in Schwann cells after NGF or IL‐17A exposure (Figure 3I,J), consistent with activation of the actomyosin contractile apparatus and cytoskeletal reorganization that could modify Schwann cell–neuron contacts and downstream signaling. Notably, siRNA‐mediated *p75NTR* knockdown partially attenuated IL‐17A or NGF‐induced p‐MLC2/MLC2 upregulation (Figure S2B,C), supporting a role for p75NTR in NGF/IL‐17A‐driven MLC2 activation in Schwann cells. Together, these data indicate that IL‐17A and NGF drive MLC2‐linked cytoskeletal remodeling in Schwann cells, a change plausibly contributing to nociceptive sensitization in psoriatic skin.

### 2.4 Schwann Cells Proliferate in Psoriatic Lesions

**FIGURE 4:**
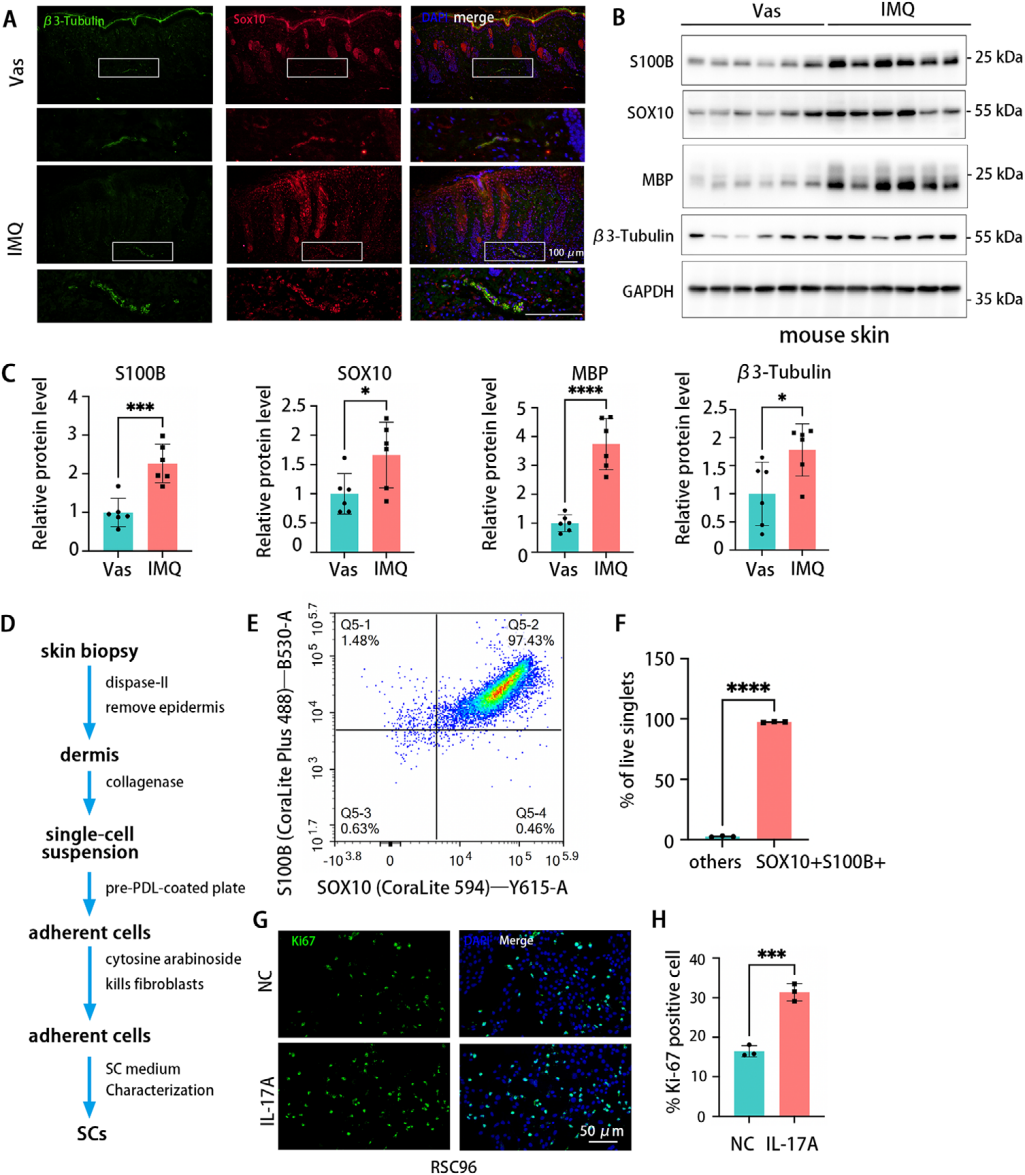
Schwann cell proliferation in psoriatic‐like lesions. (A) Representative immunofluorescence images of mouse skin stained for SOX10 (Schwann cells, red), β3‐Tubulin (nerve fibers, green), and DAPI (nuclei, blue). (B) Western blot analysis of Schwann cell‐ and nerve‐related proteins in Vas‐ and IMQ‐treated mice. (C) Quantification of S100B, SOX10, MBP, and β3‐Tubulin from B. (D) Workflow schematic for isolation of primary Schwann cells from skin. (E) Representative flow cytometry plot showing identification of Schwann cells gated as SOX10^+^S100B^+^. (F) Quantification of SOX10^+^S100B^+^ cells as a percentage of live singlets. (G) Representative immunofluorescence images of Ki67+ RSC96 cells (green) after IL‐17A stimulation (20 ng/mL); nuclei labeled with DAPI (blue). (H) Quantification of Ki67+ RSC96 cells. Scale bars as indicated. *p < 0.05, ***p < 0.001, ****p < 0.0001; unpaired Student's *t*‐test (C, F, H); *n* = 6 (C), *n* = 3 (F, H).

A specialized population of cutaneous Schwann cells at the dermoepidermal junction—termed nociceptive Schwann cells—senses mechanical stimuli and influences pain sensitivity; these cells are SOX10‐positive, and their silencing produces hyperalgesia [12]. Fluorescent IHC confirmed colocalization of SOX10^+^ Schwann cells with β3‐tubulin–labeled nerve fibers in lesional skin (Figure 4A; Figure S3A). Western blotting of IMQ lesions revealed significant upregulation of SOX10 together with other Schwann‐cell markers, including S100β and MBP (Figure 4B,C), indicating expansion of myelinating Schwann cells, non‐myelinating Remak‐bundle Schwann cells, and cutaneous nociceptive Schwann cells.

To probe function, we isolated primary human cutaneous Schwann cells (Figure 4D) and validated them by SOX10 and S100β immunofluorescence (Figure S3B) and flow cytometry (Figure 4E,F), confirming a SOX10^+^S100B^+^ Schwann cell population. The RSC96 Schwann‐cell line, commonly used to model peripheral nerve biology and neuroinflammation [33, 34], also expressed SOX10 and S100β (Figure S3C), so we used RSC96 for in vitro assays. Because IL‐17A is a dominant cytokine in the psoriatic microenvironment, we stimulated RSC96 and Schwann cells with IL‐17A to mimic lesional conditions. Ki‐67 and EdU staining revealed a marked increase in proliferating Schwann cells after IL‐17A exposure (Figure 4G,H; Figure S3D), demonstrating that IL‐17A promotes Schwann‐cell proliferation. These results show that Schwann cells expand and remodel in psoriatic lesions, supporting a role for Schwann‐cell–mediated structural and functional changes in pain sensitization.

### 2.5 Schwann Cells Attenuate NGF‐Driven DRG TrkA Signaling via p75NTR, While IL‐17 Conditioning Potentiates the Response

**FIGURE 5:**
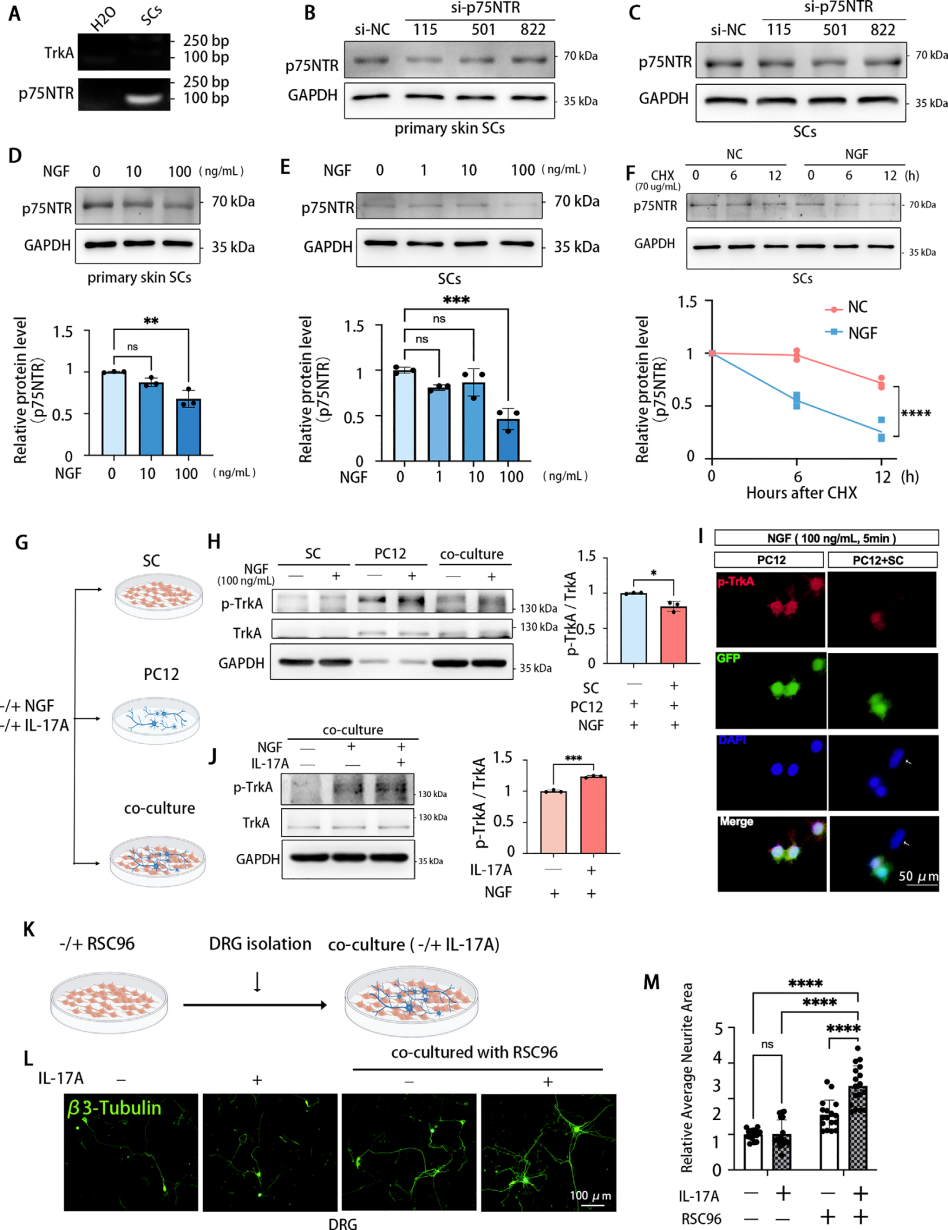
Schwann cells attenuate NGF‐Driven DRG TrkA signaling via p75NTR, while IL‐17 potentiates the response. (A) PCR and agarose gel electrophoresis analysis of *p75NTR* and *TrkA* in Schwann cells (SCs). (B, C) Validation of p75NTR knockdown in primary skin SCs and SCs using siRNA. (D, E) Western blot analysis of p75NTR expression in primary skin SCs and SCs treated with increasing NGF concentrations; accompanying quantification is shown. (F) Western blot and quantification of p75NTR in SCs treated with CHX (70 µg/mL) for the indicated times, with or without NGF (12 h). (G) Schematic of the experimental design for SCs, PC12 cells, and co‐culture under ±NGF and ±IL‐17A conditions (created using Figdraw). (H) Western blot of p‐TrkA and total TrkA in indicated cells after 5‐min NGF (100 ng/mL) stimulation; quantification of p‐TrkA/TrkA ratio shows reduced TrkA phosphorylation in the co‐culture relative to PC12 cells alone. (I) Immunofluorescence of p‐TrkA (red), GFP (green; lentiviral marker in PC12), and DAPI (blue); arrowheads indicate GFP‐negative/DAPI‐positive nuclei corresponding to SCs in the co‐culture. (J) Western blot and quantification of TrkA phosphorylation in co‐cultures treated with NGF and IL‐17A. (K) Schematic of DRG neuron and RSC96 co‐culture assay ±IL‐17A; DRG neurons cultured independently served as controls (created using Figdraw). (L) Representative immunofluorescence images of neurite outgrowth in co‐culture. (M) Quantification of average neurite area per cell. Scale bars as indicated. ns, not significant; *p < 0.05, **p < 0.01, ***p < 0.001, ****p < 0.0001; one‐way ANOVA with post hoc Tukey (D, E), two‐way ANOVA with post hoc Tukey (F, M), unpaired Student's *t*‐tests (H, J), *n* = 3 (D, E, F, H, J), *n* = 17 (M).

We next investigated whether SCs act as modulators of nociception in psoriatic skin by their responsiveness to NGF. Initial examination of NGF receptor expression revealed that TrkA mRNA was undetectable in SCs, whereas p75NTR—the canonical low‐affinity NGF receptor and SCs marker—was clearly expressed (Figure 5A). Specificity was confirmed by siRNA knockdown in both Schwann cell lines and human primary skin SCs (Figure 5B,C). NGF treatment modulated p75NTR protein in a dose‐dependent manner (Figure 5D,E); a high NGF concentration (100 ng/mL) induced a pronounced reduction in p75NTR, whereas a low concentration (10 ng/mL) had no effect. Cycloheximide (CHX) chase assays demonstrated that NGF accelerated p75NTR degradation over time, with significantly lower levels at 6 and 12 h, indicating that high NGF promotes post‐endocytic p75NTR degradation (Figure 5F).

These data suggest that under basal conditions, surface p75NTR on SCs buffers NGF, thereby attenuating neuronal activation. PC12 cells are widely used in neurobiology as a tractable model of NGF responsiveness and NGF–TrkA signaling, including neurite outgrowth–associated responses [35]. In co‐culture experiments with PC12 cells, a 5‐min NGF pulse elicited weaker TrkA phosphorylation than in monoculture (Figure 5G–I). Pre‐treatment with IL‐17A for 12 h restored and enhanced TrkA phosphorylation, indicating that IL‐17A–conditioned SCs potentiate NGF signaling (Figure 5J). Similarly, in SC–DRG co‐cultures, IL‐17A augmented the neurogenic effect of SCs, producing a significant increase in DRG neurite area (Figure 5K–M), consistent with the heightened innervation observed in psoriatic skin.

### 2.6 IL‐17A Downregulates p75NTR and Enhances NGF Expression in Schwann Cells

**FIGURE 6:**
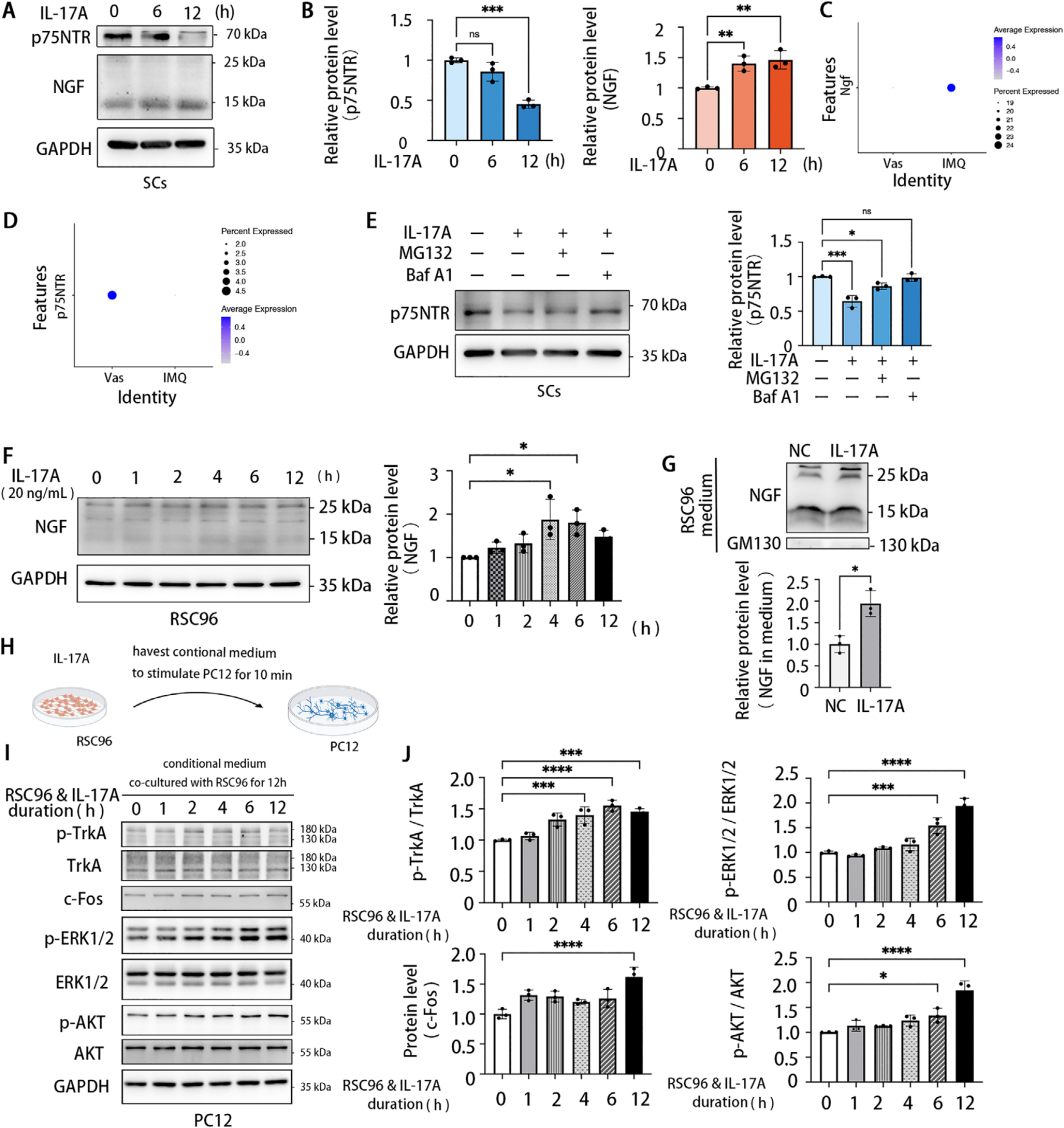
IL‐17A enhances NGF secretion and p75NTR regulation in Schwann cells. (A, B) Western blot and quantification of p75NTR and NGF in SCs stimulated with IL‐17A at the indicated time points. (C, D) Dot plots showing the expression of *p75NTR* and *Ngf* in SCs from the Vas and IMQ groups. Dot size indicates the percentage of cells expressing each gene, and dot color represents the average expression level. (E) Changes in p75NTR protein levels in SCs treated with IL‐17A (20 ng/mL) in the presence of bafilomycin A1 (Baf A1, 40 nM) or MG132 (20 µM). (F) Western blot and quantification of NGF expression in RSC96 cells treated with IL‐17A for the indicated times. (G) Western blot of NGF in supernatants of RSC96 cells stimulated with IL‐17A (20 ng/mL, 12 h); GM130 served as a negative control to confirm the absence of cellular contamination. (H) Schematic of PC12 treatment with RSC96‐derived conditioned media stimulated by IL‐17A (created using Figdraw). (I) Western blot of PC12 lysates after 5‐min exposure to RSC96‐conditioned media. (J) Quantification of protein levels in I. ns, not significant; *p < 0.05, **p < 0.01, ***p < 0.001, ****p < 0.0001; one‐way ANOVA with post hoc Tukey (B, E, F, J), unpaired Student's *t*‐test (G), *n* = 3.

We next evaluated whether psoriasis‐related mediators beyond NGF exert similar effects. IL‐17A treatment decreased p75NTR protein while increasing NGF protein in SCs (Figure 6A,B; Figure S4A). To further define the molecular features of Schwann cells in psoriasis‐like skin, we performed an integrated analysis of our previously generated scRNA‐seq datasets from Vas‐ and IMQ‐treated skin [36]. UMAP visualization identified seven major cell populations (Figure S4B). The distribution of cells from the IMQ and Vas groups is shown (Figure S4C), and the proportions of all annotated cell types are compared between the two groups (Figure S4D). Dot plots of canonical marker genes further supported the reliability of cell‐type annotations (Figure S4E). Within the Schwann cell population, *p75NTR* expression was reduced in the IMQ group, whereas *Ngf* expression levels were higher in IMQ than in Vas (Figure 6C,D).

For in vitro mechanistic validation, IL‐17A did not significantly affect *p75NTR* mRNA levels in SCs at 12 h. In contrast, a decrease was observed after 24 h, suggesting that early *p75NTR* downregulation is unlikely to result from direct transcriptional repression (Figure S4F). We therefore hypothesized that the early reduction in p75NTR protein is indirectly mediated by IL‐17A‐induced NGF upregulation. Consistent with this, exogenous NGF reduced p75NTR expression in SCs, and the NGF‐neutralizing antibody Tanezumab markedly reversed IL‐17A‐induced p75NTR downregulation (Figure S4G), indicating an NGF‐dependent effect. In addition, chlorpromazine (CPZ), which is an endocytosis inhibitor [37], blocked NGF‐induced p75NTR downregulation (Figure S4H). Because internalized plasma‐membrane proteins are commonly delivered to lysosomes for degradation in an acidic environment, we next used proteasomal and lysosomal inhibitors to define the degradation pathway. Pharmacological inhibition revealed that bafilomycin A1 (BafA1; a V‐ATPase inhibitor that blocks lysosomal acidification), but not the proteasome inhibitor MG132, blocked IL‐17A–induced p75NTR loss, implicating lysosome‐dependent endocytic degradation consistent with canonical plasma‐membrane protein turnover (Figure 6E). Concurrently, IL‐17A elevated intracellular NGF and markedly increased NGF secretion in conditioned medium (Figure 6F,G), with the Golgi marker GM130 absent from supernatants, excluding cell lysis artifacts.

Conditioned media from IL‐17A–treated SCs applied to PC12 cells for 5 min induced progressively higher TrkA phosphorylation and c‐Fos expression with increasing IL‐17A pretreatment duration (Figure 6H–J). This was accompanied by activation of the MAPK–ERK1/2 and PI3K–AKT pathways, consistent with their established roles in pain‐related neuronal activation.

### 2.7 Keap1 Downregulation Mediates IL‐17A‐Induced Nrf2 Activation and NGF Upregulation

**FIGURE 7:**
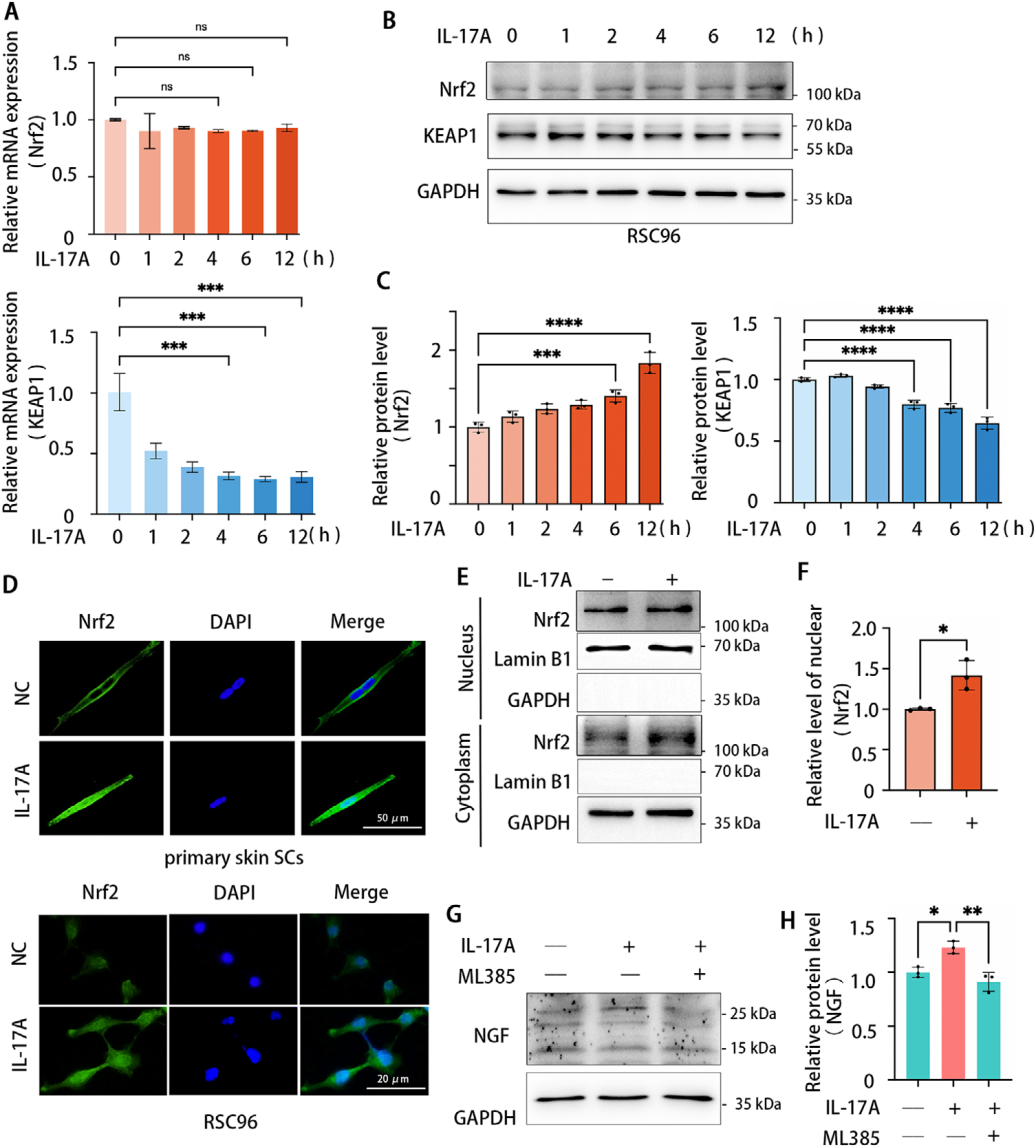
Keap1 downregulation mediates IL‐17A‐Induced Nrf2 activation and NGF upregulation. (A) mRNA expression of *Nrf2* and *Keap1* in RSC96 cells treated with IL‐17A (20 ng/mL) at indicated times. (B) Western blot analysis of Nrf2 and Keap1 protein levels in RSC96 cells under IL‐17A (20 ng/mL) treatment. (C) Quantification of protein expression from B. (D) Representative immunofluorescence images of Nrf2 (green) in RSC96 cells. (E) Western blot of nuclear and cytosolic Nrf2 in RSC96; Lamin B and GAPDH served as nuclear and cytoplasmic loading controls. (F) Quantification of nuclear Nrf2 from E. (G) Western blot of RSC96 lysates treated with IL‐17A and ML385 (5 µM). (H) Quantification of protein levels in G. ns, not significant; *p < 0.05, **p < 0.01, ***p < 0.001, ****p < 0.0001; unpaired Student's *t*‐test (F), one‐way ANOVA with post hoc Tukey (A, C, H), *n* = 3.

To elucidate the mechanism underlying IL‐17A–induced NGF upregulation, we assessed transcription factors known to regulate NGF expression. IL‐17A modestly increased the expression of multiple transcription factors (Figure S5A), with prior reports highlighting Nuclear factor erythroid 2–related factor 2 (Nrf2) as an upstream NGF regulator [38]. However, IL‐17A did not significantly alter Nrf2 mRNA (Figure 7A), suggesting post‐transcriptional regulation. Notably, Keap1, the negative regulator of Nrf2, was significantly downregulated at both mRNA and protein levels upon IL‐17A stimulation (Figure 7A,B). We further examined the transcriptional expression of Keap1 and Nrf2 within the Schwann cell population in the skin scRNA‐seq dataset. *Nrf2* showed no marked overall change between the Vas and IMQ groups (Figure S5B), whereas Keap1 was markedly reduced in the IMQ group (Figure S5C). Under basal conditions, Keap1 binds Nrf2 and promotes its ubiquitination via the Cul3‐based E3 ligase, maintaining low cytoplasmic Nrf2 [39]. IL‐17A markedly decreased Keap1 protein in RSC96 cells, coinciding with increased Nrf2 protein (Figure 7B,C), and promoted Nrf2 nuclear translocation (Figure 7D), as shown by immunofluorescence and nuclear‐cytoplasmic fractionation (Figure 7E,F). A similar time‐dependent reduction in KEAP1 protein accompanied by an increase in NRF2 protein was also observed in primary skin SCs following IL‐17A stimulation (Figure S5D,E).

These findings suggest that IL‐17A relieves Keap1‐mediated suppression of Nrf2, leading to cytoplasmic accumulation and nuclear translocation of Nrf2, which activates transcription of downstream targets, including NGF. This was confirmed using ML385, a selective Nrf2 inhibitor, which attenuated IL‐17A–induced NGF upregulation (Figure 7G,H), supporting a pivotal role for Nrf2 in mediating NGF expression downstream of IL‐17A signaling.

### 2.8 Schwann Cell–Targeted p75NTR Overexpression Attenuates IL‐17A–Evoked Nociceptive Sensitization

**FIGURE 8:**
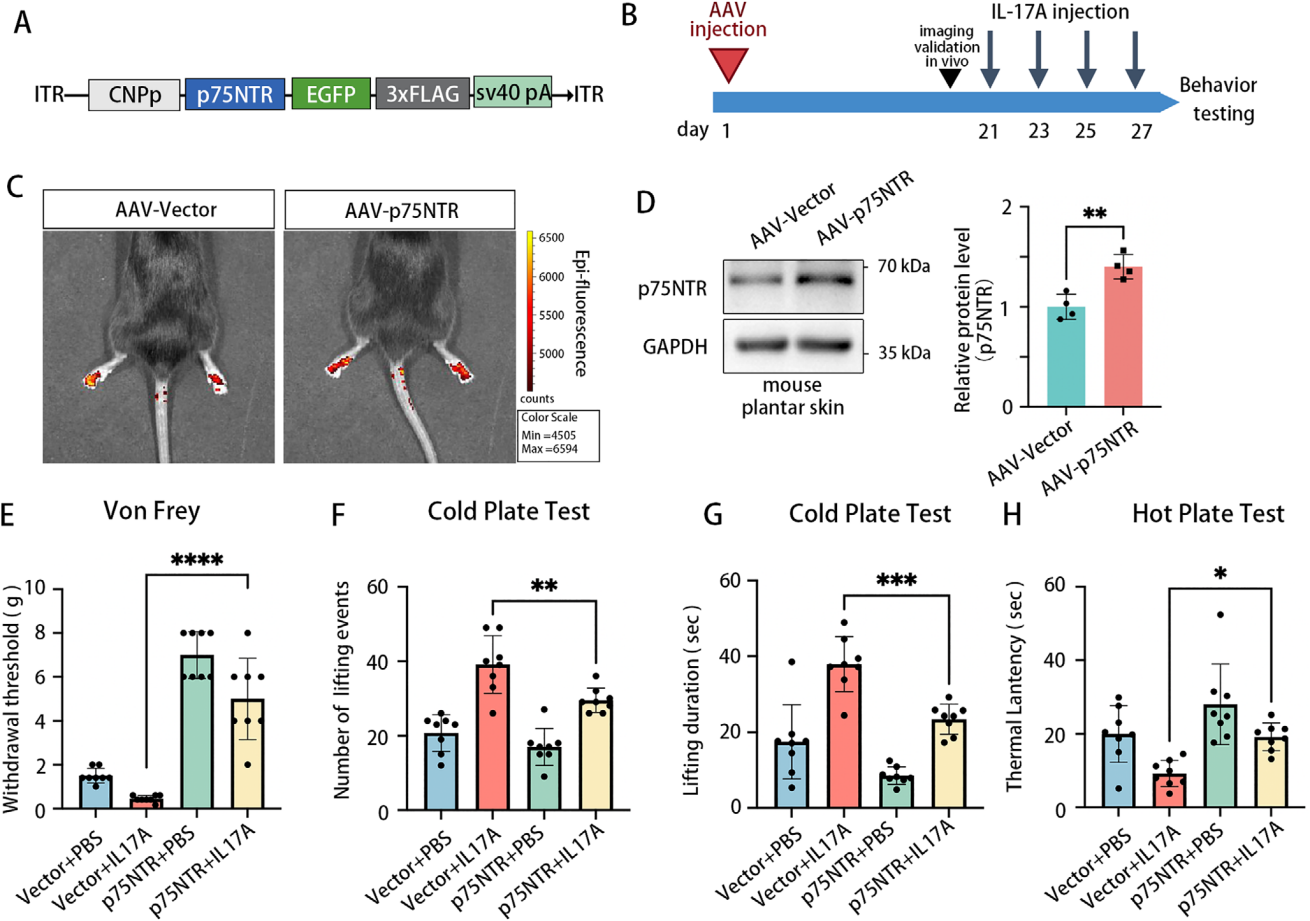
Schwann cell–targeted p75NTR overexpression attenuates IL‐17A–evoked nociceptive sensitization. (A) Schematic of the AAV expression cassette. (B) Experimental timeline: plantar AAV injection on day 1; in vivo imaging validation on day 21; repeated plantar IL‐17A (or PBS) injections on days 21, 23, 25, and 27; followed by behavioral testing. (C) Representative in vivo fluorescence imaging showing localized reporter signals at the hind‐paw injection sites in AAV‐Vector and AAV‐p75NTR mice. (D) Immunoblotting of p75NTR in mouse plantar skin lysates with GAPDH as a loading control, and quantification of relative p75NTR protein levels. (E) Mechanical sensitivity assessed by von Frey test (withdrawal threshold, g) in four groups. (F,G) Cold plate test showing the number of paw‐lifting events (F) and lifting duration (G). (H) Hot plate test showing thermal latency. *p < 0.05, **p < 0.01, ***p < 0.001, ****p < 0.0001; unpaired Student's *t*‐test (D), one‐way ANOVA with post hoc Tukey (E–H), *n* = 4 (D), *n* = 8 (E‐H).

To further examine the in vivo contribution of Schwann cell p75NTR, we locally injected a CNP promoter–driven AAV into the plantar skin to overexpress p75NTR (Figure 8A), as the CNP promoter has been used extensively to drive transgene expression in peripheral Schwann cells [40]. The experimental workflow is summarized in Figure 8B. Prior to IL‐17A administration, in vivo imaging showed focal fluorescence at the hind‐paw injection sites (Figure 8C), indicating successful transduction. Western blot analysis of plantar skin lysates further confirmed elevated p75NTR protein levels in the AAV‐p75NTR group compared with the vector control group (Figure 8D).

Mice then received repeated plantar IL‐17A injections (Figure 8B) to assess whether Schwann cell p75NTR modulates IL‐17A–evoked sensory alterations. Compared with IL‐17A–treated vector controls, mice with Schwann cell–targeted p75NTR overexpression showed a smaller IL‐17A–induced reduction in von Frey withdrawal thresholds (Figure 8E). Concordant effects were observed in thermal assays: IL‐17A–evoked nociceptive sensitization was attenuated in the AAV‐p75NTR group, as reflected by reduced cold‐plate paw lifting (events and duration) and increased hot‐plate latency (Figure 8F–H). Collectively, Schwann cell–targeted p75NTR elevation is associated with reduced IL‐17A–triggered mechanical and thermal hypersensitization.

## 3 Discussion

Clinical studies indicate that roughly one‐third of psoriasis patients experience pain in addition to pruritus [4]. Consistent with these observations, our IMQ‐induced psoriasis‐like mouse model exhibited clear pain‐associated behaviors. These mice also displayed systemic manifestations commonly associated with chronic pain, including reduced body weight and anxiety‐like behaviors, mirroring the stress, anxiety, and diminished quality of life reported in patients [41]. Together, these findings confirm that our model recapitulates key pain‐related phenotypes of psoriasis, reinforcing the clinical relevance of pain in the disease.

To investigate mechanisms underlying psoriatic pain, we focused on neuro‐immune interactions in the skin. Our data identify IL‐17A—a central cytokine in psoriasis immunopathology—as a pivotal mediator linking skin inflammation to pain sensitization. Intradermal IL‐17A injection in mouse hind paws induced psoriasis‐like epidermal changes and significantly lowered mechanical pain thresholds while increasing thermal sensitivity, reflecting cutaneous hyperalgesia. These results are consistent with reports that IL‐17 contributes to inflammatory pain and mechanical allodynia in other contexts [42].

One major mechanism by which IL‐17A enhances pain is via upregulation of NGF and its downstream signaling. NGF is a well‐established mediator of pain. Interestingly, despite upregulated NGF levels, TrkA expression was paradoxically reduced in the psoriatic skin, whereas DRG neurons exhibited elevated TrkA. This apparent discrepancy reflects NGF/TrkA retrograde signaling: NGF binding at nerve terminals induces internalization of the NGF–TrkA complex and transport to the soma, where it activates transcriptional programs that promote pain hypersensitivity [13]. These results indicate that NGF translates peripheral inflammation into sustained nociceptive signaling.

Beyond neuronal mechanisms, our study highlights the contribution of Schwann cells to psoriatic pain. Traditionally viewed as axon‐supporting glia, Schwann cells actively participate in sensory signaling. In psoriasis models, we observed Schwann cell proliferation and remodeling in inflamed skin. Transmission electron microscopy revealed increased Schwann cell nuclei, tighter axonal enwrapment, and Schwann cell–axon complexes at the dermal‐epidermal junction in IMQ‐treated and IL‐17A‐injected mice, paralleling structures seen in patient biopsies. IL‐17A or NGF treatment induced Schwann cells to adopt a flattened, spread morphology with increased cell area, while total F‐actin remained unchanged, indicating cytoskeletal reorganization rather than polymerization. Such remodeling may enhance Schwann cell–axon interactions.

Our results further reveal that IL‐17A modulates Schwann cell function to modulate TrkA‐related pain signaling. Schwann cells expressed p75NTR but not TrkA, and IL‐17A decreased p75NTR protein while dramatically increasing NGF production and secretion. Under homeostatic conditions, p75NTR may likely acts as an NGF buffer, limiting nociceptor activation. IL‐17A disrupts this mechanism, enhancing NGF availability and TrkA activation on sensory fibers. In addition, NGF itself may directly promote p75NTR degradation in Schwann cells, whereas under IL‐17A stimulation, this process appears to be at least partly dependent on the increase in endogenous NGF produced by Schwann cells. Based on the inhibitor and neutralization experiments, we speculate that IL‐17A‐induced p75NTR downregulation is primarily mediated by NGF‐dependent receptor internalization, followed by lysosomal degradation.

At the molecular level, IL‐17A acts via the Keap1–Nrf2 pathway. IL‐17A downregulated Keap1, enabling Nrf2 stabilization and nuclear translocation. While Nrf2 is classically associated with antioxidant responses, it also drives growth factor transcription, including NGF [43]. In Schwann cells, IL‐17A‐induced Keap1 downregulation integrates inflammatory and stress‐response pathways to enhance NGF production, contributing to psoriatic pain.

Notably, the relationship between inflammatory cytokines and NGF appears to be highly context dependent. In several inflammatory settings, cytokines such as TNF‐α, IL‐1β, and IL‐6 have been shown to enhance NGF production or potentiate NGF‐associated nociceptive signaling, supporting the concept that inflammatory cues can engage neurotrophic pathways to amplify pain [44]. Consistent with this broader framework, our findings suggest that IL‐17A may similarly reshape Schwann cell signaling by elevating NGF and facilitating p75NTR turnover. At the same time, opposite effects have also been described in certain cellular contexts, indicating that the direction and magnitude of NGF regulation likely depend on the inflammatory milieu, responding cell type, and downstream signaling state [45]. In addition, heterogeneity in the degree of inflammation among psoriatic lesions may lead to differences in the levels of IL‐17A and NGF within the perineural microenvironment, thereby resulting in variation in the buffering function of Schwann cell p75NTR. This difference may, at least in part, explain why not all psoriatic lesions in patients are accompanied by pain.

Although this study was based on psoriasis‐like models, the mechanism described here may also be relevant to other chronic pain conditions characterized by persistent peripheral inflammation, increased NGF signaling, and neurotrophin imbalance, such as osteoarthritis, rheumatoid arthritis, and postherpetic neuralgia [46, 47]. Notably, clinical efforts targeting the NGF pathway have already demonstrated analgesic potential [48]. For example, the anti‐NGF monoclonal antibody tanezumab has been shown to improve pain and physical function in chronic osteoarthritis pain [47]. Therefore, targeting Schwann‐cell state or restoring the local balance between NGF and p75NTR may represent a more precise adjunct therapeutic strategy for chronic inflammatory pain, although its translational feasibility and safety remain to be further investigated.

This study still has several limitations. First, although our single‐cell RNA‐sequencing analysis of skin from Vas/IMQ‐treated mice provided support for our conclusions, we also recognize that the sample size of the single‐cell dataset needs to be further expanded to enable a more in‐depth exploration of the functional heterogeneity of Schwann cell subpopulations. Second, although we validated the relationship between changes in p75NTR in Schwann cells and cutaneous sensation at the cellular level, as well as in IMQ‐induced, IL‐17A–induced, and AAV‐intervened mouse models, further clinical studies are still needed to investigate the association between Schwann cells and sensory abnormalities in patients with psoriasis, given the complexity of human disease and the subjective variability in pain perception.

**FIGURE 9:**
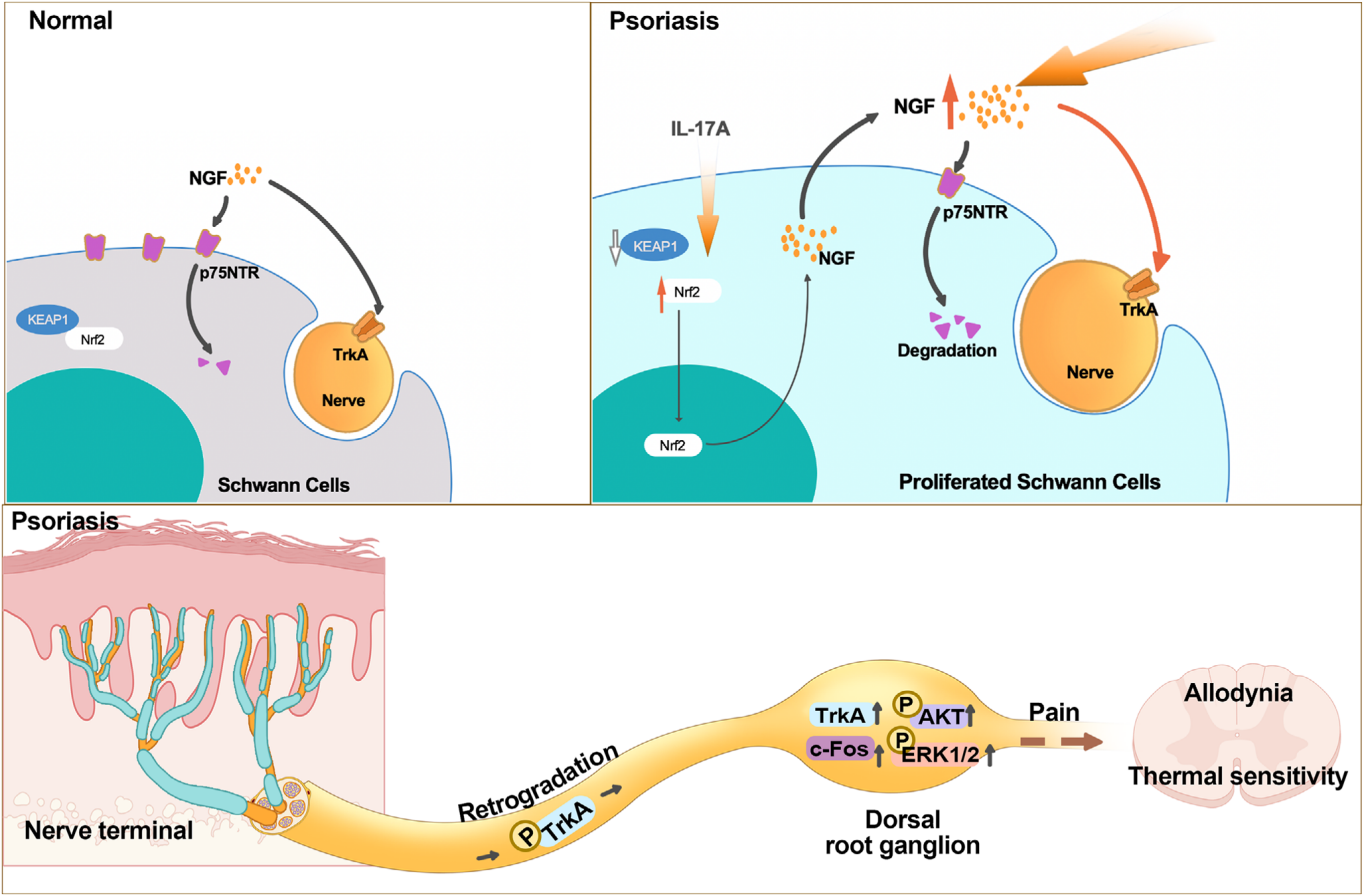
Schematic of depletion of p75NTR in Schwann cells driven by inflammation mediates cutaneous pain in psoriasis. Schwann cells limit NGF signaling of cutaneous peripheral nerves through the receptor p75NTR. However, inflammation driven by interleukin‐17A raised NGF levels, which led to degradation of p75NTR, triggering TrkA internalization and retrograde transport and thereby potentiating pain‐related signaling in DRG neurons.

In summary, our study suggests that Schwann cells in psoriatic lesions are important participants in the regulation of cutaneous sensation. Our findings indicate that, in an inflammatory milieu, IL‐17A promotes NGF production in Schwann cells while reducing p75NTR levels, thereby potentially weakening the NGF‐buffering effect of p75NTR and influencing sensory nerve–related signaling (Figure 9). Accordingly, Schwann cells may exhibit a dual potential under different conditions, acting either to limit or to promote pain signaling. These findings highlight a neurogenic dimension of psoriasis and suggest that Schwann cell–centered IL‐17A–NGF–p75NTR regulation may contribute to the development and progression of psoriatic pain.

## 4 Experimental Section

### 4.1 Human Subjects

All procedures involving human skin biopsies were approved by the Biomedical Research Ethics Committee Involving Human Subjects of Shandong Provincial Hospital Affiliated to Shandong First Medical University (SYLPD‐NSFC: No. 2022–149). Written informed consent was obtained from all donors. Skin biopsies for immunofluorescence imaging were collected from treatment‐naïve psoriasis patients and healthy control donors undergoing surgery, in accordance with the Declaration of Helsinki.

### 4.2 IMQ/IL‐17A‐Induced Mouse Models

Female C57BL/6J mice (6–8 weeks old) were obtained from the Shandong Academy of Medical Sciences. For the IMQ‐induced psoriasis model, mice (n = 6) received daily topical applications of 62.5 mg 5% IMQ cream (#H20030129, Med‐shine Pharma) to shaved dorsal skin for six consecutive days; controls received Vaseline cream. Mice were euthanized on day 7.

For the IL‐17A‐induced model, recombinant mouse IL‐17A (#HY‐P73174, MCE) was injected intradermally into hind paws at 1 µg per paw in 20 µL PBS, administered on days 1, 3, 5, and 7. Control mice received PBS alone. Recombinant AAV‐injection driven by the *CNP* promoter was used to overexpress p75NTR in mouse Schwann cells (GeneChem, Shanghai, China). Under isoflurane anesthesia, 20 µL of viral suspension was slowly injected into the plantar skin of the hind paw using a microsyringe, and control mice received an equal volume of control vector. Subsequent experiments were performed 3 weeks after viral injection. All procedures complied with the NIH Guide for the Care and Use of Laboratory Animals were approved by Experimental Animal Ethics Committee of Shandong Provincial Hospital Affiliated to Shandong First Medical University (SYDLP‐NSFC: No. 2022–149).

### 4.3 Open Field Test

Exploratory behavior, anxiety‐like behavior, and locomotor activity were assessed in a white acrylic arena (45 × 45 × 40 cm). Mice were placed individually at the center, and total distance traveled, number of center entries, and time spent in the central 20 × 20 cm zone were recorded over 10 min under 100 lux illumination. Analysis was performed using ANY‐MAZE software.

### 4.4 Von Frey Test

Mice were acclimated on a metal grid for 40 min per day for two days prior to testing. Mechanical sensitivity was assessed using von Frey filaments (0.008–300 g; Exacta) applied to the plantar surface, beginning with a 0.08 g filament. A rapid paw withdrawal within 4 s was considered a response. Each filament was tested five times, and a positive response required withdrawal in at least three trials. Investigators were blinded to treatment groups.

### 4.5 Cold/Hot Plate Test

Thermal pain sensitivity was measured using a Hot/Cold Plate device (SA‐YLS‐21A, BioMed Instruments). Mice were acclimated in a restrainer on the plate at room temperature for 5 min per day for two consecutive days. For cold sensitivity, the plate was set to 4°C; cumulative hind paw lifts and total duration over 5 min were recorded. For heat sensitivity, the plate was set to 55°C, and latency to jump or lift both hind paws was recorded immediately.

### 4.6 Human Skin NGF ELISA

Human skin tissues were weighed after removal of residual surface blood and subcutaneous fat and homogenized in pre‐chilled PBS at a ratio of 90 µL PBS per 10 mg tissue. Samples were thoroughly homogenized on ice using a tissue homogenizer to avoid heat‐induced protein degradation. The homogenates were then centrifuged at 10,000–12,000 × *g* for 10 min at 4°C, and the supernatants were collected for subsequent analysis. NGF levels were measured using a Human NGF ELISA Kit (ABclonal, RK10077) according to the manufacturer's instructions. Optical density (OD) values were recorded after addition of standards and samples, and NGF concentrations in skin homogenates were calculated from the standard curve and normalized to tissue weight, expressed as NGF content per mg of skin tissue.

### 4.7 Transmission Electron Microscopy

Tissues were rinsed in PBS and fixed in 3% glutaraldehyde (pH 7.4), trimmed to 1 × 1 × 3 mm, rinsed, post‐fixed in 1% osmium tetroxide, dehydrated through graded acetone, infiltrated with Epon 812, and embedded. Semi‐thin sections were followed by ultrathin sectioning (70–100 nm) using an LKB ultramicrotome (LKB‐V), stained with uranyl acetate and lead citrate, and imaged on a JEM‐1200EX transmission electron microscope (JEOL Ltd) using a CCD camera (MORADA‐G2, Olympus).

### 4.8 H&E Staining and Fluorescent Immunohistochemistry

Paraffin‐embedded human and mouse skin sections (3–5 µm) were deparaffinized, rehydrated, and subjected to antigen retrieval and blocking. Endogenous peroxidase activity was quenched, and sections were incubated overnight at 4°C with primary antibodies. For H&E staining, hematoxylin and eosin were applied sequentially with standard differentiation and fixation. For fluorescent IHC, a universal SP kit (SP90000, ZSGB‐Biotech) was used. Primary antibodies included SOX10 (1:200, sc‐365692, Santa Cruz), β3‐Tubulin (1:100, #5566, CST), and NGF (1:100, MA5‐32067, Invitrogen). Secondary antibodies were CoraLite488‐conjugated Goat Anti‐Rabbit IgG (H+L) (1:500, SA00013‐2, PTG) and CoraLite594‐conjugated Goat Anti‐Mouse IgG (H+L) (1:500, SA00013‐3, PTG).

### 4.9 Cell Culture and Treatment

Human Schwann cells (hTERT ipn02.3 2λ, ATCC CRL‐3392) and RSC96 rat Schwann cells (FH1100, Fuheng, Shanghai, China) were cultured in DMEM (KGM12800N‐500, KeyGEN) supplemented with 10% FBS (#A5670701, Gibco) and 1% penicillin–streptomycin. PC12 cells (FH0413, Fuheng) were cultured in RPMI‐1640 (KGL1501‐500, KeyGEN) with 10% horse serum (#3079058, Gibco), 5% FBS, and 1% penicillin–streptomycin. Cells were maintained at 37°C with 5% CO_2_ and passaged at 70%–80% confluence. Human IL‐17A protein (HY‐P70527, MCE), Rat IL‐17A protein (HY‐P78556, MCE), human β‐NGF (HZ‐1222, Proteintech), Tanezumab (HY‐P99221, MCE), ML385 (HY‐100523, MCE) and CPZ (HY‐12708, MCE) were used for cell stimulation as specified in the experiments. siRNAs targeting human *p75NTR* (*NGFR*) and a negative control siNC were synthesized commercially. The siRNA sequences were as follows:

Si*p75NTR*‐115: sense, 5’‐CAGCGUGAGUGCUGCAAA (dTdT)‐3’; antisense, 5’‐UUUGCAGCACUCACCGCUG (dTdT)‐3’.

si*p75NTR*‐501: sense, 5’‐CCGUGUGCGAGGACACCGA (dTdT)‐3’; antisense, 5’‐UCGGUGUCCUCGCACACGG (dTdT)‐3’.

si*p75NTR*‐822: sense, 5’‐GGAACAGCUGCAAGCAGAA (dTdT)‐3’; antisense, 5’‐UUCUGCUUGCAGCUGUUCC (dTdT)‐3’.

### 4.10 Primary Schwann Cell Isolation from Human Skin Tissue

The procedures were performed according to previously described methods [49]. T25 culture flasks were pre‐coated with poly‐L‐lysine at 37°C for 3 h and rinsed twice with 1× DPBS. Dermal tissue was rinsed in DMEM, minced, and digested in 2 mg/mL collagenase in DMEM (5 mL) at 37°C for 2.5 h with gentle trituration every 30 min until fully dissociated. The resulting suspension was filtered through a 70 µm cell strainer, diluted with 5 mL complete DMEM to quench collagenase, and centrifuged at 870 × *g* for 5 min at room temperature. Pelleted cells (a mixed population of fibroblasts and Schwann cells) were resuspended in complete DMEM, counted for viability, and seeded onto PLL‐coated T25 flasks at 4.0 × 10^3^ cells/mL in 5 mL medium per flask. After overnight incubation (12–16 h, 37°C, 5% CO_2_), non‐adherent cells were removed, and cultures were washed three times with DPBS. Fibroblasts were selectively depleted by treating cultures with 10 µM cytosine β‐D‐arabinoside in complete DMEM for 24 h. After three washes with DPBS, Schwann cells were maintained in Schwann cell complete medium (1701, ScienCell), with medium changes every other day until reaching ∼ 80% confluence.

### 4.11 Flow Cytometry

Primary human skin‐derived Schwann cells were dissociated and collected as single‐cell suspensions. After washing with PBS, the cells were fixed and permeabilized (562574, BD). The cells were then subjected to intracellular staining with antibodies against SOX10 (CL594‐66786, Proteintech) and S100B (CL488‐15146, Proteintech), with corresponding isotype controls included for gating reference. After staining, samples were acquired on an Agilent NovoCyte flow cytometer. Debris and abnormal events were excluded based on forward and side scatter profiles, and SOX10 and S100B expression was further analyzed within the single‐cell population. The proportion of SOX10^+^S100B^+^ cells among single cells was used to define the Schwann cell population.

### 4.12 Immunofluorescence (IF) Staining

Cells were plated onto glass coverslips in 6‐well plates. After treatment, cells were fixed with 4% paraformaldehyde for 15 min and permeabilized with PBS containing 0.5% Triton X‐100 for 20 min. Non‐specific binding was blocked with normal goat serum (cw0130s, CWBIO) for 1 h at room temperature. Cells were then incubated overnight at 4°C with primary antibodies: NGF (1:100, MA5‐32067, Invitrogen), SOX10 (1:200, sc‐365692, Santa Cruz), S100B (1:200, 15146‐1‐AP, Proteintech), β3‐Tubulin (1:100, #5566, Cell Signaling Technology), p‐TrkA (1:200, #9141, Cell Signaling Technology), Ki67 (1:200, 27309‐1‐AP, Proteintech), and Nrf2 (1:200, 16396‐1‐AP, Proteintech). After washing with PBS, cells were incubated for 1 h at room temperature with fluorescent‐conjugated secondary antibodies. Secondary antibodies: CoraLite488‐conjugated Goat Anti‐Mouse IgG (H+L) (SA00013‐1, Proteintech), CoraLite488‐conjugated Goat Anti‐Rabbit IgG (H+L) (SA00013‐2, Proteintech), CoraLite594‐conjugated Goat Anti‐Mouse IgG (H+L) (SA00013‐3, Proteintech), or CoraLite594‐conjugated Goat Anti‐Rabbit IgG (H+L) (SA00013‐4, Proteintech), as appropriate. F‐actin was labeled with CoraLite Plus 488‐conjugated Phalloidin (PD00001, Proteintech), and nuclei were counterstained with DAPI (ab104139, ABcam). Images were captured using an Olympus BX63F microscope, and quantification was performed with ImageJ software. Cell morphology was assessed by calculating the ratio of the maximum to minimum cell diameter, where a value of 1 represents a perfectly round cell.

### 4.13 Quantitative Real‐Time PCR

Total RNA was extracted using a cell RNA kit (Sparkjade, #AC0205) and reverse‐transcribed to cDNA using a cDNA synthesis kit (CWBIO, #CW2569M) according to the manufacturer's instructions. qPCR reactions were performed using SYBR Green PCR master mix (CWBIO, #CW0957). Primer sequences are listed in Table S1.

### 4.14 DRG Neuron Isolation

DRG neurons were harvested from 6–8‐week‐old female C57BL/6 mice. Under a stereomicroscope (SZX7, Olympus), DRGs were dissected and placed in D‐Hanks solution (H1045, Solarbio) on ice. Tissue was digested in DMEM containing 0.1 mg/mL DNase I (DN25, Sigma‐Aldrich), 0.4 mg/mL trypsin type I (T8003, Sigma‐Aldrich), and 1 mg/mL collagenase type IA (C9891, Sigma‐Aldrich) at 37°C for 35 min to generate single‐cell suspensions. Cells were cultured in Neurobasal medium (21103049, Gibco) supplemented with B‐27 (17504044, Gibco), 2 mM L‐glutamine (25030081, Gibco), 5% FBS, and 1% antibiotic–antimycotic solution.

### 4.15 Tissue Protein Extraction

Mouse skin tissues were processed using a total protein extraction kit for skin tissue (Invent, #SA‐01‐SK). Dissociated DRG neurons were lysed in RIPA buffer (CWBIO, #CW2333S) supplemented with protease inhibitors (HY‐K011, MedChemExpress) immediately after dissociation. Cell membranes were disrupted via ultrasonication, and protein lysates were mixed with SDS‐PAGE loading buffer for immunoblot analysis.

### 4.16 Western Blotting Analysis

Cells were washed with PBS and lysed in RIPA buffer containing protease inhibitors. Lysates were mixed with SDS‐PAGE loading buffer (Solarbio, #P1016) and boiled at 98°C for 5 min. Cytoplasmic and nuclear fractions were separated using a Cytoplasmic and Nuclear Extraction Kit (Invent, SC‐003). Proteins were separated by SDS‐PAGE, transferred to PVDF membranes (Millipore, #ISEQ00010), and blocked with 5% BSA in PBST (0.05% Tween‐20) for 1 h at room temperature. Membranes were incubated overnight at 4°C with primary antibodies (Anti‐S100B (1:1000, sc‐393919, Santa Cruz), anti‐SOX10(1:1000, sc‐365692, Santa Cruz), anti‐MBP (1:1000, sc‐271524, Santa Cruz), anti‐β3‐Tubulin (1:1000, #5566, Cell Signaling Technology), anti‐p75NTR(1:1000, ab52987, ABcam), anti‐NGF(1:1000, A14216, Abclonal), anti‐GM130 (1:1000, #70767, Cell Signaling Technology), anti‐IL‐17A (1:1000, 13082‐1‐AP, Proteintech), anti‐p‐TrkA (1;1000, #9141, Cell Signaling Technology), anti‐TrkA (1:1000, #2505, Cell Signaling Technology), anti‐c‐Fos (1:1000, #2250, Cell Signaling Technology), anti‐p‐ERK1/2 (1:1000, #4370, Cell Signaling Technology), anti‐ERK1/2 (1:1000, #9102, Cell Signaling Technology), anti‐p‐AKT (1:2000, #4060, Cell Signaling Technology), anti‐AKT (1:1000, #9272, Cell Signaling Technology), anti‐GAPDH (1:5000, 60004‐1‐Ig, Proteintech), anti‐KEAP1(1:1000, 10503‐2‐AP, Proteintech), anti‐Nrf2 (1:1000, #20733, Cell Signaling Technology), anti‐pMLC2(1:1000, #95777, Cell Signaling Technology) and anti‐MLC2(1:2000, 10906‐1‐AP, Proteintech), followed by 1 h of incubation at room temperature with HRP‐conjugated secondary antibodies (goat anti‐mouse IgG, 1:5000, SA00001‐1, Proteintech; goat anti‐rabbit IgG, 1:5000, SA00001‐2, Proteintech). Protein bands were visualized using a chemiluminescence detection kit (PK10002, Proteintech).

### 4.17 Single‐Cell Analysis

The single‐cell RNA‐seq data analyzed in this study were obtained from our group's previously published work and are available in the Gene Expression Omnibus (GEO) under the sample accession numbers GSM5795800, GSM4547483, GSM5024748, GSM5024749, GSM5795802, GSM4547481, GSM4547482, GSM5024746, and GSM5024747.

Single‐cell transcriptomic sequencing data were analyzed using the Seurat R package (v4.2.0). Briefly, the raw expression matrix was imported to create Seurat objects, followed by quality control (QC) filtering based on sequencing metrics; samples containing fewer than 1,000 cells were excluded. To mitigate batch effects across samples, data integration and batch correction were performed using the Harmony algorithm. Dimensionality reduction and visualization were then conducted with UMAP based on the top 30 principal components. Finally, cell clustering was performed using the “FindNeighbors” and “FindClusters” functions. In mice, the gene symbols corresponding to *p75NTR* and *Nrf2* are *Ngfr* and *Nfe2l2*, respectively.

### 4.18 Cell Type Annotation

Cell types were annotated according to the expression of established marker genes, including endothelial cells (*Pecam1*, *Emcn*, *Cldn5*, *Esam*, *Cdh5*), fibroblasts (*Col1a1*, *Col1a2*, *Lum*, *Dcn*, *Col3a1*), keratinocytes (*Krt10*, *Krt1*, *Krt14*, *Krt15*, *Krt5*), macrophages (*Fcgr1*, *Basp1*, *H2‐Ab1*, *Adgre1*, *Itgam*), mesenchymal cells (*Prrx1*, *Serpine2*, *Twist1*, *Snai1*, *Snai2*), Schwann cells (*Plp1*, *Sox10*, *S100b*, *Cnp*, *Cryab*), and T cells (*Cd3e*, *Cxcr6*, *Trdv4*, *Cd3d*, *Cd3g*).

### 4.19 Statistical Analysis

All data are presented as mean ± SD. Statistical analyses were performed using GraphPad Prism 10. Where applicable, data were normalized to the corresponding control group. For comparisons between two groups, unpaired Student's *t*‐test was used. For comparisons among multiple groups, one‐way or two‐way ANOVA followed by Tukey's multiple‐comparisons test was indicated in the corresponding figure legends. A *p‐*value < 0.05 was considered statistically significant. The sample size (*n*) for each experiment is indicated in the corresponding figure legends.

## Author Contributions

Y.W., S.H., and N.D. conceived and designed the study. Y.W., L.X., and Z.Y. developed the methodology. Y.W., L.X., C.P., R.C., P.Z., and X.L. performed the investigation. Y.W. and L.X. carried out the visualization. S.H. and N.D. supervised the study. N.D. provided funding Y.W., S.H., and N.D. wrote the original draft of the manuscript. Y.W., L.X., Q.H., S.H., and N.D. reviewed and edited the manuscript.

## Conflicts of Interest

The authors declare no conflicts of interest.

## Supporting information




**Supporting File**: advs75064‐sup‐0001‐SuppMat.docx.

## Data Availability

The data that support the findings of this study are available from the corresponding author upon reasonable request.
