# Supplementary material for: Depletion of p75NTR in Schwann Cells Driven by Inflammation Mediates Cutaneous Pain in Psoriasis
Source: Adv Sci (Weinh). 2026 Apr 3;13(34):e23189. doi: 10.1002/advs.202523189 (PMC13285172; doi:10.1002/advs.202523189)
Supplement: Supplementary file 1 — Supporting File: advs75064‐sup‐0001‐SuppMat.docx. [file ADVS-13-e23189-s001.docx]

Supporting Information

**Depletion of p75NTR in Schwann Cells Driven by Inflammation Mediates Cutaneous Pain in Psoriasis**

Yibo Wang, Linlin Xu, Chenglong Pan, Ruonan Cao, Piao Zeng, Xinxing Lyu, Qingxia Hu, Zhenzhen Yan, Shuhong Huang, Ningning Dang*


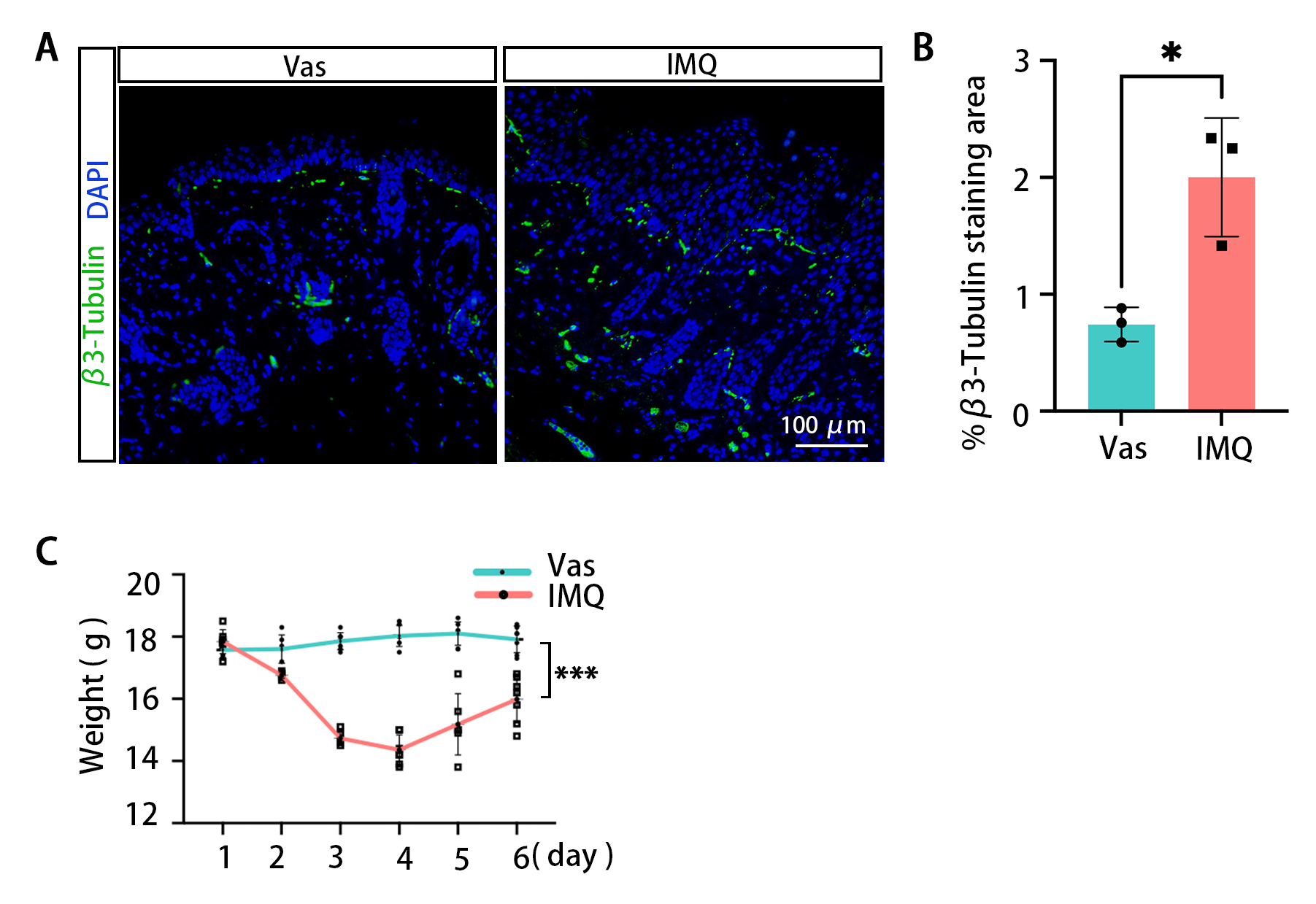


Fig. S1. Psoriasis Evokes Pain-associated Coping Behaviors

(A) Representative immunofluorescence images showing β3-tubulin staining in skin from Vas- and IMQ-treated mice. (B) Quantification of the percentage of β3-tubulin-positive staining area in (A). (C) Line graph illustrating mean body weight changes over the 6-day treatment period. Scale bars as indicated. **P* < 0.05, ****P* < 0.001; unpaired Student’s *t*-test (B); two-way ANOVA with Tukey’s post hoc test (C). n = 3 (B), n = 6 (C).


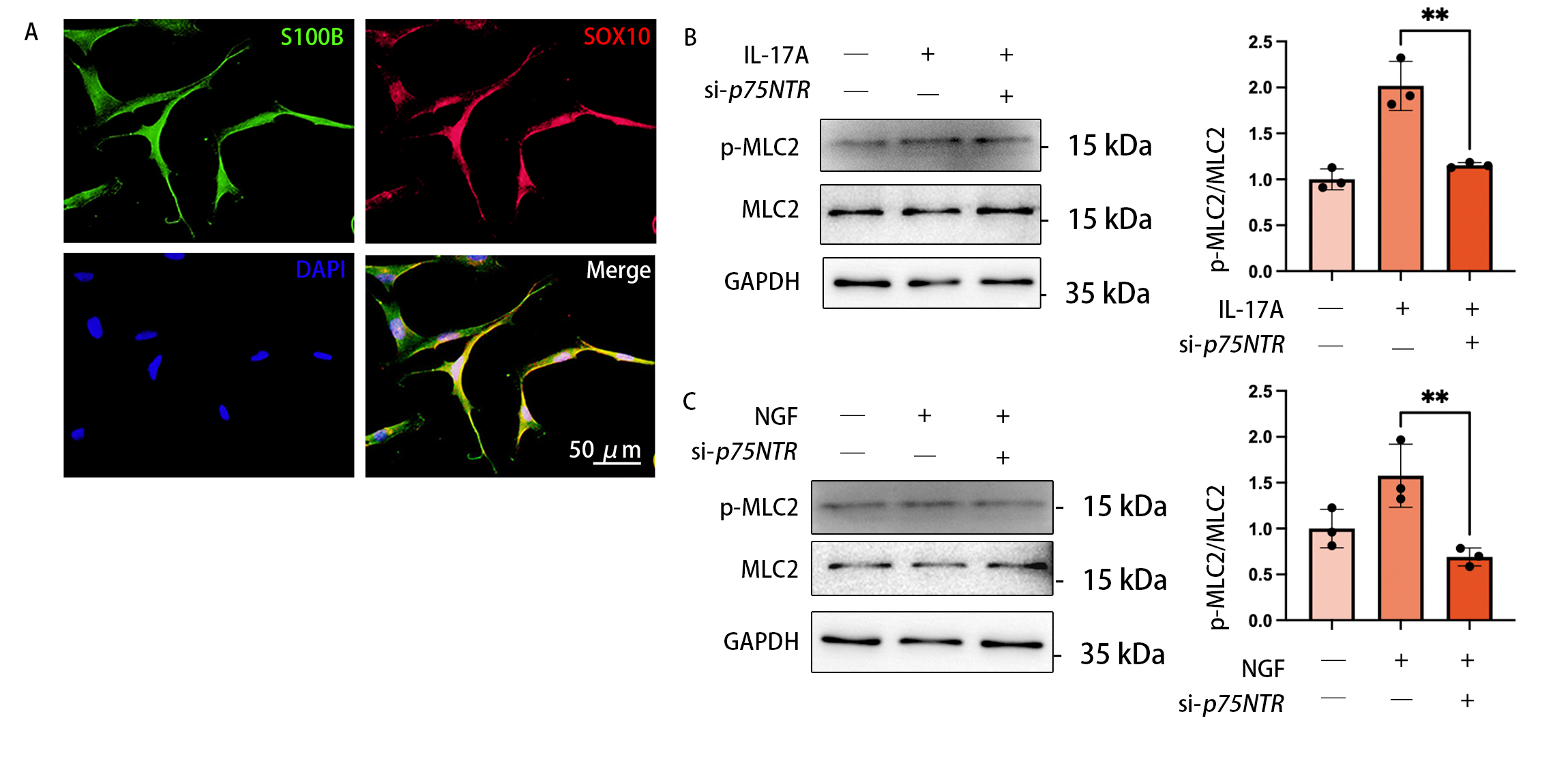


Fig. S2 *p75NTR* knockdown attenuates IL-17A- or NGF-induced MLC2 phosphorylation in Schwann cells.

(A) Schwann cells were confirmed by immunofluorescence staining for the characteristic markers S100B (green) and SOX10 (red). Nuclei were counterstained with DAPI (blue). (B, C) Schwann cells transfected with si-*p75NTR* were stimulated with IL-17A or NGF. Levels of p-MLC2 and total MLC2 were assessed by western blot, with quantification of the p-MLC2/MLC2 ratio shown. Scale bars as indicated. ***P* < 0.01; one-way ANOVA with Tukey’s post hoc test (B, C). *n* = 3.


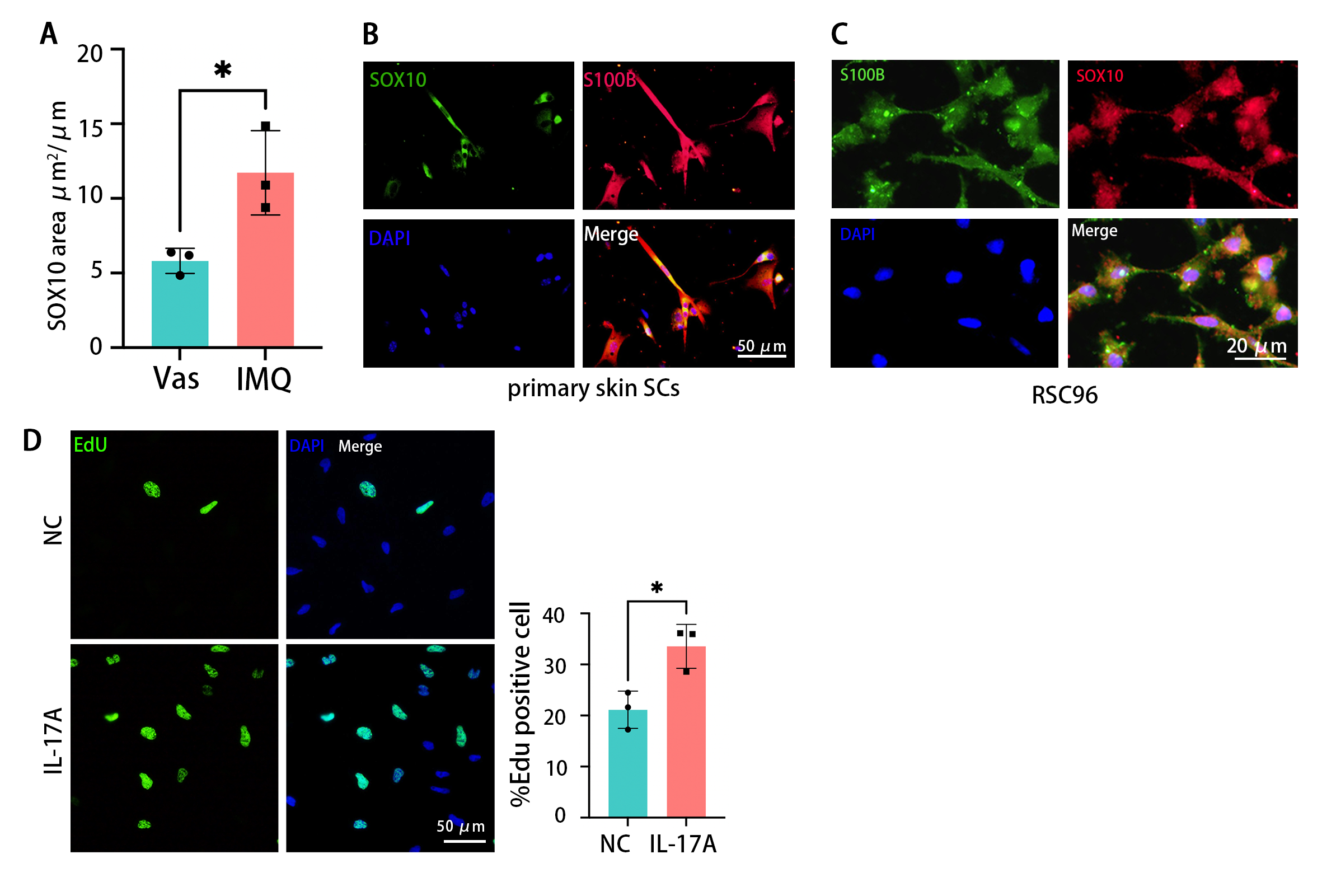


**Fig. S3 Schwann cell proliferation in psoriatic-like lesions**

(A) Quantification of the mean SOX10-positive area surrounding nerve fibers in skin sections from Vas and IMQ groups. (B) Marker characterization of primary skin Schwann cells, SOX10 (green) and S100B (red). (C) Marker characterization of cultured RSC96 cells, S100B (green) and SOX10 (red). (D) Representative EdU staining (green) with DAPI (blue) and corresponding quantification in Schwann cells treated with IL-17A or negative control. Scale bars as indicated. **P* < 0.05; unpaired Student’s *t*-test (A, D). *n* = 3.


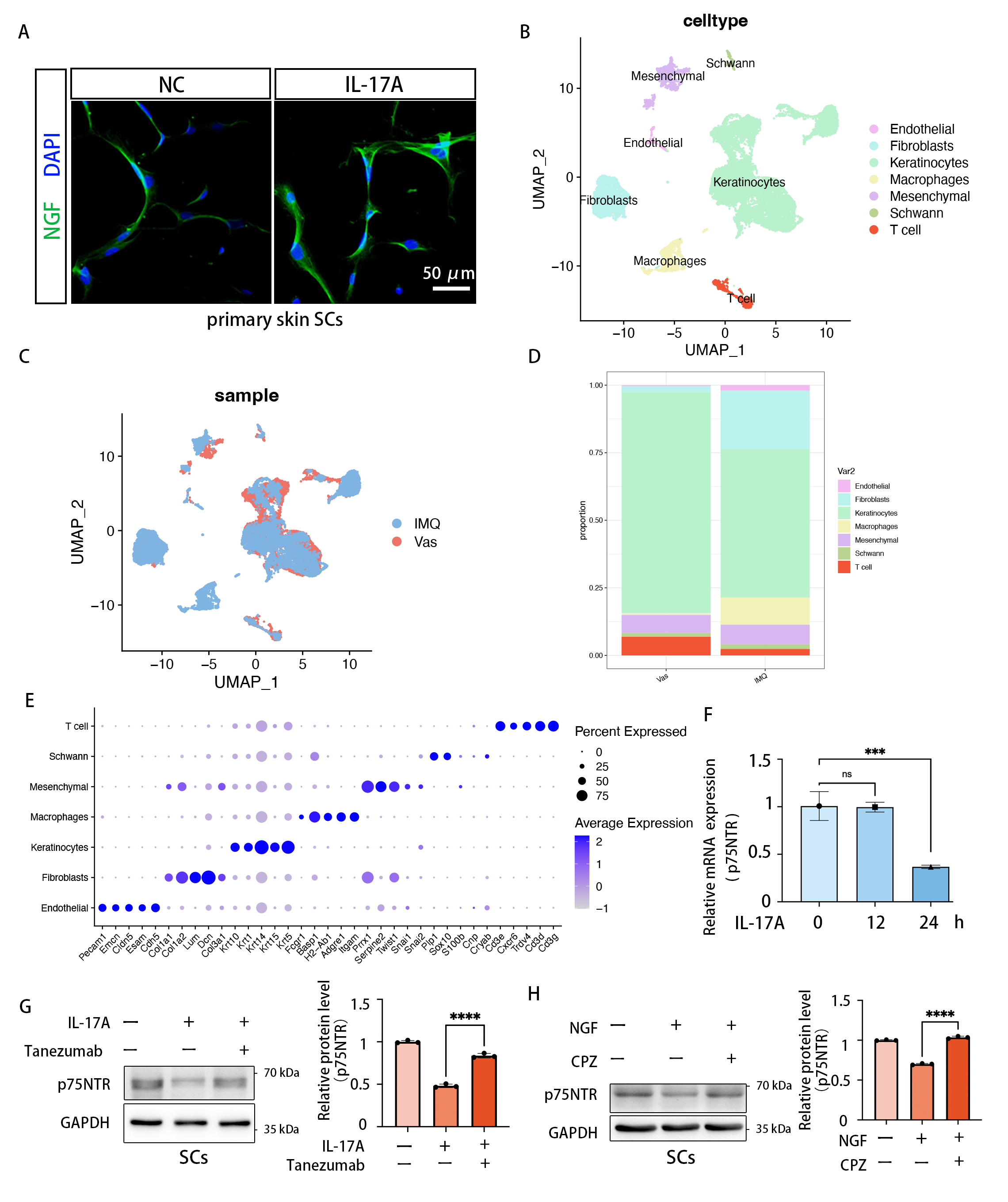


**Fig. S4 IL-17A Enhances NGF Secretion and Regulates p75NTR in Schwann Cells**

(A) Representative immunofluorescence images showing NGF expression in primary skin SCs treated with PBS or IL-17A. NGF is shown in green and nuclei are counterstained with DAPI (blue). Scale bar, 50 μm. (B, C) Single-cell atlas of skin from wild-type and psoriatic mice. UMAP projection of 20,709 skin cells from two samples, colored by cell type (B) and experimental group (C). Each dot represents a single cell. (D) Relative proportions of major skin cell types in the Vas and IMQ groups. (E) Dot plot displaying expression of cell type–specific marker genes; dot size reflects the proportion of cells expressing each gene and dot color indicates average expression. (F) qRT-PCR analysis of p75NTR mRNA levels in Schwann cells following IL-17A stimulation. (G) Representative western blots and quantification of p75NTR protein levels in SCs treated with IL-17A (20 ng/mL) with or without tanezumab (100 ng/mL). (H) Representative western blots and quantification of p75NTR protein levels in SCs treated with NGF in the presence or absence of CPZ (10 μM). ns, not significant; ****P* < 0.001, *****P* < 0.0001; one-way ANOVA with Tukey’s post hoc test (F–H). *n* = 3.

**
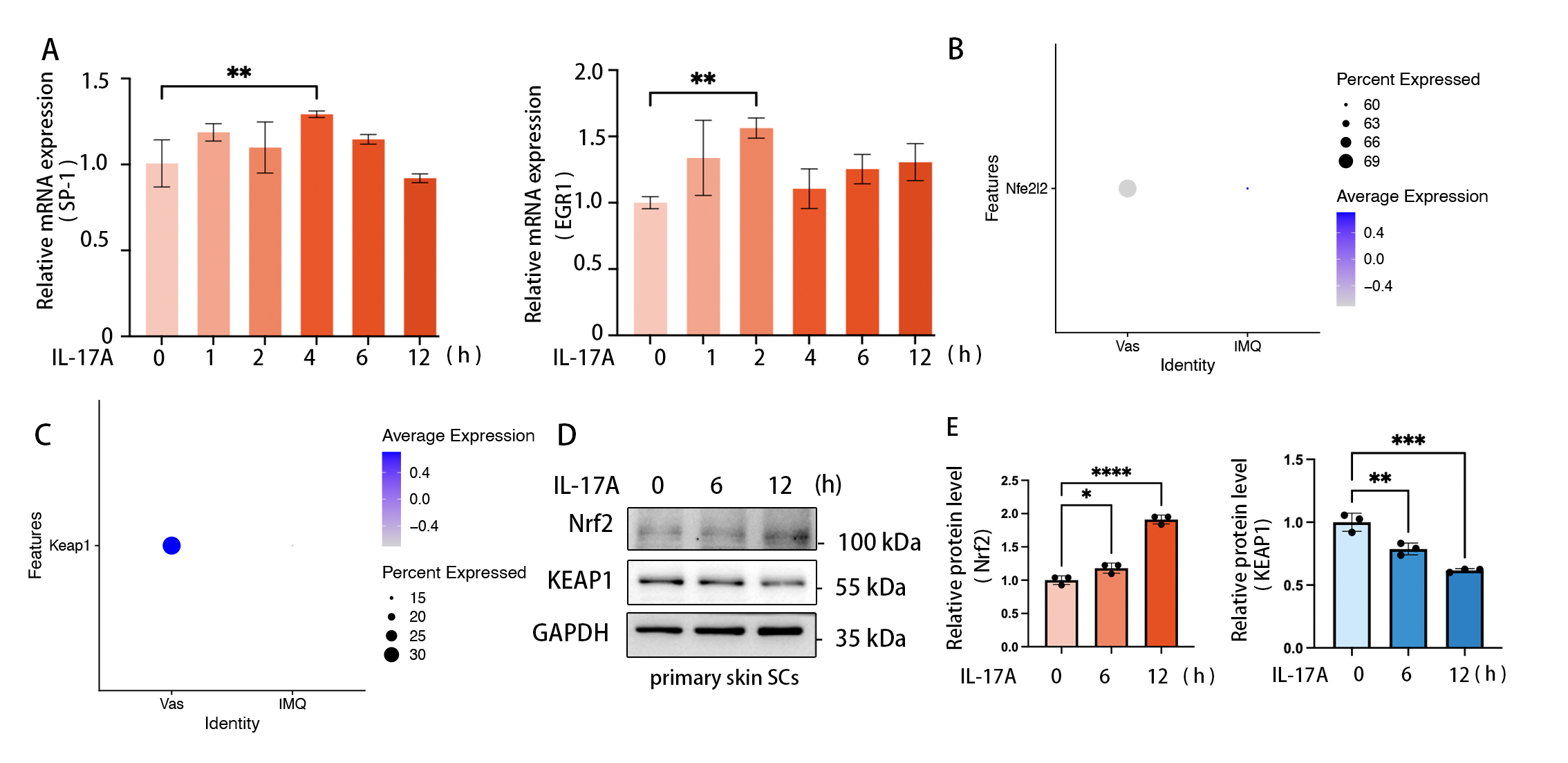
**

**Fig. S5 Keap1 Downregulation Mediates IL-17A-Induced Nrf2 Activation and NGF Upregulation**

(A) mRNA expression of *SP-1* and *EGR1* in RSC96 cells following IL-17A treatment at the indicated time points. (B, C) Dot plots showing expression of Nrf2 (Nfe2l2) and Keap1 in Schwann cells from the Vas and IMQ groups. Dot size represents the proportion of cells expressing each gene, and dot color indicates mean expression level. (D) Representative western blot images showing Nrf2 and KEAP1 protein levels in primary skin SCs treated with IL-17A for the indicated durations. (E) Quantification of Nrf2 and KEAP1 protein levels in SCs following IL-17A treatment for 0, 6, and 12 hours. **P* < 0.05, ***P* < 0.01, ****P* < 0.001, *****P* < 0.0001; one-way ANOVA with Tukey’s post hoc test (A, E). *n* = 3.

Table S1. Primers used in this study.

| **Target** | **Sequence** |
| --- | --- |
| *TrkA* forward | CTGTACCCCCGATCTTGACG |
| *TrkA* reverse | TGAAGGAGAGATTCAGGCGAC |
| *p75NTR* forward | AGGCCTGTACACACACAGC |
| *p75NTR* reverse | GAGAACGTCACGCTGTCCA |
| *c-FOS* forward | CGGGTTTCAACGCCGACTA |
| *c-FOS* reverse | TGGCACTAGAGACGGACAGAT |
| *Nrf2* forward | GTCAGCTACTCCCAGGTTGC |
| *Nrf2* reverse | GGCAAGCGACTGAAATGTAGG |
| *KEAP1* forward | ATGCACTTCGGGGAGGAGGA |
| *KEAP1* reverse | GGGCAGTCGTATTTGACCCA |
| *SP-1* forward | TGCCACCATGAGCGACCAA |
| *SP-1* reverse | GGGTGACTCAATTCTGCTGC |
| *EGR1* forward | CTGCAGATCTCTGACCCGTT |
| *EGR1* reverse | ATGTCAGAAAAGGACTCTGTGGT |
| *GAPDH* forward | CACCATCTTCCAGGAGCGAG |
| *GAPDH* reverse | CTCGTGGTTCACACCCATCA |
